# Wastewater Quality Estimation through Spectrophotometry-Based Statistical Models

**DOI:** 10.3390/s20195631

**Published:** 2020-10-01

**Authors:** Daniel Carreres-Prieto, Juan T. García, Fernando Cerdán-Cartagena, Juan Suardiaz-Muro

**Affiliations:** 1 Department of Mining and Civil Engineering, Universidad Politécnica de Cartagena, 30202 Cartagena, Spain; 2 Department of Information and Communications Technologies, Universidad Politécnica de Cartagena, 30202 Cartagena, Spain; fernando.cerdan@upct.es; 3 Department of Electronic Technology, Universidad Politécnica de Cartagena, 30202 Cartagena, Spain; juan.suardiaz@upct.es

**Keywords:** LED spectrophotometer, wastewater pollutant characterization, organic matter, suspended solids, nutrients

## Abstract

Local administrations are increasingly demanding real-time continuous monitoring of pollution in the sanitation system to improve and optimize its operation, to comply with EU environmental policies and to reach European Green Deal targets. The present work shows a full-scale Wastewater Treatment Plant field-sampling campaign to estimate COD, BOD_5_, TSS, P, TN and NO_3_^−^N in both influent and effluent, in the absence of pre-treatment or chemicals addition to the samples, resulting in a reduction of the duration and cost of analysis. Different regression models were developed to estimate the pollution load of sewage systems from the spectral response of wastewater samples measured at 380–700 nm through multivariate linear regressions and machine learning genetic algorithms. The tests carried out concluded that the models calculated by means of genetic algorithms can estimate the levels of five of the pollutants under study (COD, BOD5, TSS, TN and NO_3_^−^N), including both raw and treated wastewater, with an error rate below 4%. In the case of the multilinear regression models, these are limited to raw water and the estimate is limited to COD and TSS, with less than a 0.5% error rate.

## 1. Introduction

Urban sanitation systems involve sewer networks (SNs) and wastewater treatment plants (WWTPs). Integrated and joint management of these is mandatory to overcome issues arising from stormwater runoff episodes which, in short periods, overload the system in terms of pollutants and flowrates as well as those coming from the dry-weather daily variation of pollution load. Rainfall runoff collected and conveyed through combined sewers has an important influence on the efficiency of the entire treatment process [1,2,3,4]. Urban sanitation systems must comply with EU policies to halt the deterioration in the status of EU water bodies and the environment: Water Framework Directive (WFD, 2000/60/EC), Groundwater Directive (GWD, 2006/118/EC), Environmental Quality Standards Directive (EQS, 2008/105/EC), Directive 91/271/EEC or the Urban Wastewater Directive (UWWTD), Directive 2006/7/EC or the EU Bathing Water (Directive 2006/7/EC) and Marine Strategy Framework Directive (Directive 2008/56/EC). Continuous, real-time, reliable information about the pollutants in the input sewage is of great interest to improve and optimize the operation of the sanitation systems, to fulfill EU environmental policies and to reach European Green Deal targets [5].

Developing sensors for the continuous monitoring of wastewater parameters is a major scientific and technical challenge due to the variability of wastewater characteristics as well as the extreme physical-chemical conditions the sensors are subjected to [6,7]. Optical techniques including UV–Vis spectroscopy and near-infrared spectroscopy NIR have been used to reliably characterize solids, organic matter and nitrates in wastewater for over a decade [8,9,10,11,12,13,14,15,16,17,18,19,20,21,22,23,24,25]. UV–Vis refers to the interaction between samples and radiation in the 200–780-nm wavelength range at single or multiple wavelengths to estimate a number of parameters [19]. It is fast, non-destructive and environment-friendly since it does not require chemicals to be added. It is coupled with multivariate data analysis such as partial least squares (PLS) regression to generate a regression model based on spectral data to estimate the water quality parameters [8,26,27,28]. Several studies have shown good agreement in online continuous monitoring of chemical organic demand (COD) using UV–Vis spectroscopy [8,11,16,17,18,20,22,23,24,25]. Total suspended solids (TSS) has also been predicted through UV–Vis and NIR [11,17,18,22,23,24]. Nitrates (NO_3_^−^N) achieved results with UV–Vis with an error of ~25% and correlation coefficients of 0.87 [24]. Other works have presented the second derivative UV–Vis spectroscopy absorption spectrum for NO_3_^−^N calibration [10]. There are continuous sensors and analyzers capable of operating online with UV spectrophotometry that can be used to monitor nitrate and nitrite concentration in water samples [14,22]. Promising and long-term measurements have been developed in several cities, namely Linz [21], Graz, Ecully and Vienna [23], addressing online UV–VIS sensors for long-term sewer monitoring.

Statistical techniques have become necessary tools to establish correlations between optical sensors signals and the continuous monitoring of wastewater quality. Linear regression (LR) and other machine learning techniques such as support vector machine (SVM), evolutionary algorithm method (EVO) and artificial neural networks (ANNs) have been used for the mathematical treatment of spectral absorbance patterns to estimate five-day biochemical oxygen demand (BOD_5_) and chemical oxygen demand (COD) values of wastewater samples [11,12,13,17,19,25]. From the absorbance response curves measured in an extensive range of wavelengths, specific measured values are used and combined through statistical techniques to generate a relation between pollutant concentration and absorbance or transmittance. The slope transmittance calculation and other mathematical operations such as the second derivative are also used for the estimation of biochemical loads [10,15,27].

Deploying spectroscopic-based sensors throughout the sewerage system to monitor the pollution load in real time requires an enormous amount of equipment that must therefore meet the requirement of being cost-effective. The literature already describes the availability of compact and low-cost UV–Vis spectrophotometers to monitor WWTP processes [29,30,31,32,33,34,35,36,37,38,39,40]. In addition, the installation of storm water storage and sedimentation tanks is usually economically unacceptable, thus a monitoring system to optimize the management of the sewer network is the most cost-effective and, probably, the most ecological variant as well [16]. Despite this scenario, the number of online studies remains relatively limited due to certain drawbacks such as the variability of sample composition and other matrix effects (particle size and moisture content) that complicate the absorbance response correlation [19].

The present work shows a full-scale WWTP study for the estimation of chemical oxygen demand (COD), biological oxygen demand at five days (BOD5), total suspended solids (TSS), phosphorus (P), total nitrogen (TN) and nitrate nitrogen (NO_3_^−^N) by means of site-specific multivariate linear regressions (MLR) and machine learning genetic algorithms (GA) from the absorbance and transmittance in the UV–near visible and visible 380–700 nm wavelength range. A campaign of around 1200 analytical determinations in the lab was carried out in the Cabezo Beaza WWTP (Region of Murcia, Spain), during the period from June 2019 to April 2020. The samples were collected from the Influent Wastewater (Raw water) and Effluent treated water of the WWTP. They consisted of six classes of contaminant analysis (COD, BOD5, TSS, P, TN and NO_3_^−^N). Each class had a size of approximately 200 samples. About half of the samples corresponded to the input of the WWTP (raw water) and the rest to the output (treated water). The equipment used for the transmittance characterization in the UV–near visible and visible range is cost-effective, own developed and has been previously calibrated [29]. This is an offline research study, considered as the first step to reaching a continuous and online monitoring system, at sanitation-system scale, which allows assisting in the control of the pollutants that reach the treatment plant, as well as contributing to the improvement of the treatment processes carried out.

The rest of the article is organized as follows: Section 2shows all the materials and methods used for the development of the research work. It describes the characteristics of the experimental campaign carried out, indicating the conditions for data collection, the number of samples analyzed and the polluting parameters under study. It also includes a description of the equipment developed for the process of characterizing the samples, as well as the different calculation procedures used to obtain the models for estimating the pollutant load from the spectrophotometric data. In Section 3, the characteristics of the analyzed water, both raw and treated, are described, as well as the different models for the estimation of the pollutant load obtained by the multivariable linear regression models, as well as the genetic algorithm. The results and comparisons of the models are also presented. Finally, Section 4 discusses the considerations reached at the end of the research work.

## 2. Materials and Methods

### 2.1. Experimental Campaign

Samples were collected in the waterline of the Cabezo Beaza WWTP at two different sampling points, in the period June 2019 to April 2020:

- Influent wastewater at the entrance of the WWTP: raw water
- Treated water, at the exit of the secondary settler, prior to the third treatment: secondary wastewater

Responding to the requirements of the inspection sampling campaigns by the supervisory administration (Wastewater Administration of Murcia Region, ESAMUR), the samples were integrated, i.e., they were taken homogeneously during 24 h in a 5-L volume, by means of an accumulated sample of 200 mL/h. After this, they were collected around 7:00 AM daily and tested almost simultaneously. Once in the laboratory of the plant, the samples used in the present research were not pre-treated through any filtering process, with the intention of reproducing the conditions of automatic sampling for the continuous monitoring sensors. Tests at the WWTP lab were in correspondence with Standard Methods (SM) and International Organization for Standardization (ISO), as described in Table 1. Standard methods were developed by members of the Standard Methods Committee (SMC) with the mutual publication of the American Public Health Association (APHA), American Water Works Association (AWWA) and the Water Environment Federation (WEF).

To develop the statistical models to estimate the pollutant load from the spectrophotometric data between 380 and 700 nm, it was necessary to obtain two datasets for each of the samples: the input data, based on the spectrophotometric analysis of the samples, and the analytical values of the pollutant load measured by the WWTP’s laboratory (output data).

### 2.2. Spectrophotometric Device

**Figure 1:**
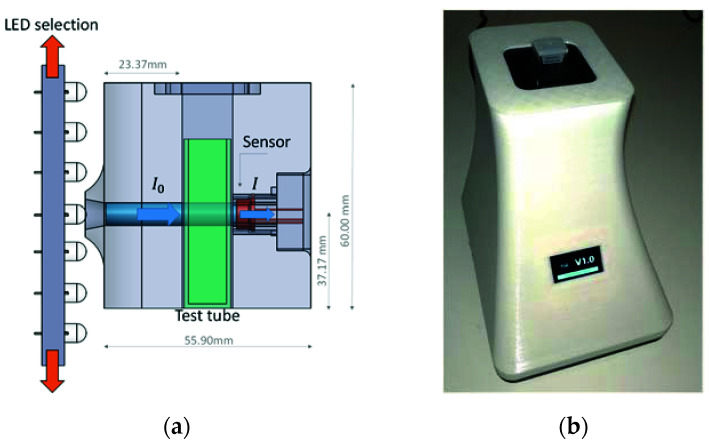
View of the developed LED spectrophotometry equipment: (**a**) schematic view of the assembly; and (**b**) equipment developed for spectrophotometric analysis.

Figure 1 shows a schematic view of the spectrophotometry equipment based on LED technology (Figure 1a) and an image of the equipment (Figure 1b), which we developed to analyze the spectral response of wastewater samples. This device was previously calibrated with a commercial spectrophotometer in the UV–near visible and visible wavelength range 380–700 nm and the results were presented in previous research [29]. This is a cost-effective piece of equipment which, to reduce its size and to improve its portability, uses no optical element such as lenses, diffraction matrix, or monochromators. The interior of the proposed assembly was constructed entirely in black thermoplastic PLA with a 3D printer, while the outer casing is made of white PLA, although the color of the casing does not affect the operation of the device.

From the results of the research conducted in [29] on the use of LED technology, the device can model 81 wavelengths within 380–700 nm using only 33 limited-bandwidth LEDs. To select the working LED, the equipment has a motorized system consisting of a panel that slides vertically, which has all the light-emitting diodes, so that they can be aligned with the sample being analyzed.

The light from the LED passes through the sample via a 6-mm-diameter channel to the sensor [30]. The sensor S1223, whose accuracy was previously studied, was chosen for the analysis of the samples [29,31]. The tests carried out revealed that the most accurate results were obtained when the sensor (Figure 1a, right) was as close as possible to the sample without touching it, and the light source (Figure 1a, left) [32,33,34,35,36,37,38,39,40] was at a distance of about 23.77 mm with regard to the test tube. All samples were stored in standard 12 mm × 12 mm × 50 mm plastic test tubes of the SEOH brand [41], designed for spectrophotometry purposes.

### 2.3. Regression Models

#### 2.3.1. Multivariate Linear Regression

A multivariate linear regression (MLR) model is proposed where the entire evaluation of the transmittance and absorbance spectra within 380–700 nm is used. As input variables, both the transmittance and the absorbance values obtained by the 81 wavelengths supported by the developed equipment were used, giving rise to 162 variables.

To be able to validate the models calculated with data not used during their collection, the data were divided into two groups: training data (for the development of the models) and test data (for the validation of the models). These data were divided at random with proportions of 66% and 34%, respectively.

The MLR model was developed with the IBM SPSS Statistics software, using a model fitting based on partial least squares [42]. A prior step to the calculation of any model is to determine the existence of outliers. A box and whiskers diagram was built to determine the existence of outliers and subsequently eliminate them. To make a multivariate model, the data must follow a normal distribution, i.e., that the P-value is greater than 0.1 (90% confidence interval) calculated with the Kolmogorov–Smirnov and Shapiro–Wilk tests. If it does not follow a normal distribution, the Box–Cox transformation [43] should be carried out.

Once the data follow a normal distribution, SPSS tools are used to perform the analysis, selecting the “Stepwise” [44] calculation option, which allows for more optimized calculation models. Therefore, the wavelengths (regressors) present in each of the MLR models were automatically selected by SPSS according to the following methodology.

The process starts by introducing the regressor whose P-value is highest within the range 0.05 and 0.1 (input and output criteria, respectively). In the following interaction, SPSS reintroduces the regressor with the highest *p*-value (within the range) and then reevaluates the model to check if any of the regressors introduced are no longer significant and/or there is multicollinearity in the model, i.e., that there are regressors correlated with each other in the model. This process is repeated with all possible combinations of regressors.

Once SPSS has calculated the models, those whose coefficient of determination R-square ($\bar{R^{2}}$) is greater are selected and a check is made to ensure that the model does not include correlated variables, by checking that the Variance inflation factor is less than 7.

#### 2.3.2. Genetic Algorithms

Another statistical technique used in the present work is the genetic algorithm. This was developed to calculate correlation models between the input variables (spectrophotometric data) and output variables (contaminating parameters) of water samples.

Within the category of genetic algorithm, a type of model known as “symbolic regression” was implemented, which is a type of regression analysis that seeks the space of mathematical expressions to find the model that best fits a certain dataset. The calculation model was developed in Python using the following libraries: TensorFlow [45], NumPy [46,47] and gpLearn [48]. Before processing the data, outliers were removed using Box and Whisker analysis for the response variable. Symbolic regression works through a system of “trees” composed of interconnected nodes. Each of these nodes can be composed of a variable (transmittance and absorbance values for each of the 81 wavelengths, i.e., 162 variables) or operators/functions (addition, subtraction, division, multiplication, trigonometric functions, etc.)

The process of finding a model that correlates the input variables with the output variables is based on an evolutionary process. As a starting point, we used both multivariate linear regression models calculated for the pollutant parameters as well as randomly initialized functions based on certain restrictions of length and type of operators. This evolutionary model has 100 generations, where 1000 different trees are generated in each generation, with a mutation rate of 15% by the subtree swapping method [49,50,51,52,53,54]. Each tree is generated starting with an addition node, from which a random number of nodes are derived, which can be constants, variables or operations. The nodes consisting of operations will have new descending nodes, which can once again be constants, variables or operations. The branching process of the tree continues until all the terminations are constant or variable or the total length and/or depth of the tree is exceeded. Each randomly generated tree is tested with the input data (absorbance/transmittance) in order to check how close the response variable (the pollutant load, e.g., COD) is to the values calculated by the WWTP. The trees closest to this result will be mutated (combined) to generate another 1000 trees, and the process is repeated until 100 generations are completed.

Each time the model calculation process is started, the GA takes the training data at random in the first iteration and based on that selection calculates the model. To guarantee the validity of the models presented, a cross-validation process was carried out, consisting of the repeated execution of the model generation process, in order to obtain different estimation models for each parameter. In all cases, the models presented a very similar level of accuracy, although their mathematical expressions (coefficients) were different. Therefore, in the present manuscript, only one of the multiple calculation models is shown, since all of them are equally valid.

It is important to point out that the data were subjected to the same process of detection and elimination of outliers described in Section 2.3.1 for MLR before being analyzed.

The symbolic regression (genetic algorithm), is based on the development of a neural network, which must be trained and tested. As training data, we used 66% of the input data, taken at random, while the remaining data were used to test the validity of the calculated models, also taken at random.

This ratio was chosen according to the design criteria in [55,56], which recommend a ratio of 70%:30% when dividing the data. However, to achieve a more generalist model, that is, one that does not depend so much on the input data used, we decided to use the ratio 66%:34%.

The formulas generated by the algorithm can have a variable extension, include all types of operations, both arithmetic and trigonometric, exponential, or logarithmic functions, as well as more or fewer parameters.

#### 2.3.3. Model Comparison

To be able to make a comparison between the two types of models (MLR and GA), several parameters were calculated: the Root-Mean-Square Deviation (RMSD) [57] and the error index, Er, through Equations (1) and (2):

(1) $$RMSD=\sqrt{\frac{1}{n}\sum_{i}^{n}{\left(X_{reference_{i}}-X_{Estimated_{i}}\right)}^{2}},$$

(2) $$Er\left(\%\right)=\frac{\sum_{i}^{n}\left(X_{reference_{i}}-X_{Estimated_{i}}\right)}{\sum_{i}^{n}X_{reference_{i}}}*100,$$

where *n* is the number of samples; and $X_{reference}$ and $X_{Estimated}$ are the values of the polluting parameters (COD, BOD_5_, TSS, P, TN and NO_3_^−^N) obtained by the analytical methods used by the wastewater treatment plant and by the calculation models, respectively. It is necessary to point out that negative error value denote that the calculated models tend to provide lower than expected estimates, as opposed to positive values.

### 2.4. Data Platform

To enable the relations between the transmittance/absorbance data provided by the LED-Spectrophotometer and pollutant parameters measured by the wastewater treatment plants to be determined, all the information generated has been stored in a single website [58], so that it can be easily downloaded in CSV format for further analysis.

**Figure A1:**
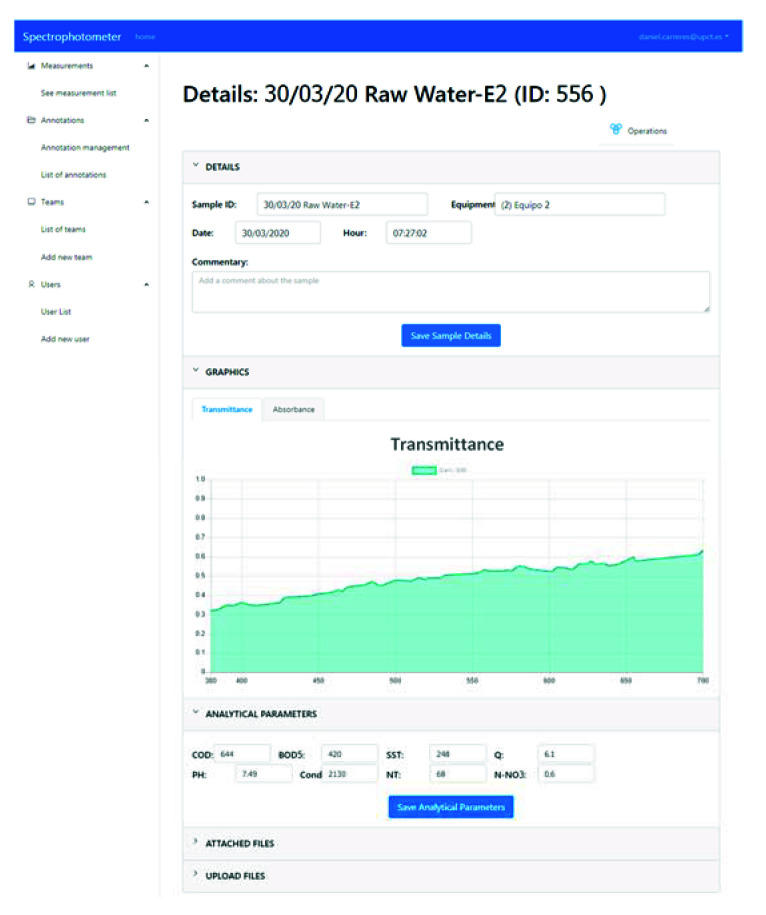
Web platform for data storage. Details of a stored sample (Wastewater. Raw Water).

Each of the stored samples contains information on the date and time of its analysis, identification of the equipment used to measure it, identification of the wastewater plant that carried out the analysis, and the spectrophotometric data and polluting parameters calculated by the treatment plants. Figure A1 (Appendix A) shows a view of the web platform for data storage.

### 2.5. Comparison with Commercial Equipment

Due to the difficulty of carrying out real-time analysis of wastewater quality, many researchers have developed analysis systems based on indirect measurements of the pollutant load, such as turbidity.

Systems such as those presented in [59,60] are able to carry out the analysis of turbidity of samples through the use of LED technology and low-cost photosensors. The equipment described in [59] consists of a probe that allows the measurement of transmittance and lateral light scattering generated by a set of LEDs of different wavelengths, using two broad-spectrum photodiodes. This allows them to measure the degree of opacity of water samples with great precision, in addition to measuring other parameters such as chlorophyll, which is very useful for analyzing water quality.

Other equipment, such as those presented [61,62], go a step further and combine turbidity analysis with other sensors that allow measuring the amount of nitrates, dissolved oxygen, or conductivity of the samples, among others. These parameters are very useful when trying to know the water quality in a fast way and in real time.

This research work sought to develop a simpler system, where external sensors are not required to carry out an analysis of water quality, in order to obtain a smaller and cheaper equipment. To do this, unlike previous systems that make use of measurements of the turbidity of the samples, that is, one or a small number of wavelengths, the system presented in this research work determined, from a wider range of wavelengths (380–700 nm), the values of COD, BOD5, TSS, P, TN and NO_3_^−^N with a high precision and without the need to rely on external parameters such as conductivity or temperature.

In contrast to the previous systems, it is worth mentioning the s::can’s [63] system. This system is capable of analyzing multiple parameters of contaminants from the spectral response of water samples taken in real time, in a similar way to the system presented in this research work. However, although this equipment is capable of generating a wider emission spectrum, it is based on xenon lamps. These lamps have high energy consumption and require the use of diffraction gratings to diffract the light beam before reaching the CCD sensor, which are responsible for its almost 500-mm length. This also increases the cost of the equipment and significantly increases its dimensions. The equipment developed in this research work is based on the use of LED diodes, where, as was verified in previous works [29], the use of optical elements is not required to function.

## 3. Result and Discussion

### 3.1. Transmittance Characterization and Sampling Analysis

A wastewater plant carries out analyses at different points in its treatment process to check how the treatment process is working. At each of these points, the pollutant load changes, as do the biological matter and inorganic particles present in the water, which react at certain wavelengths. Spectrophotometric analysis can also be used to observe these variations at each point in a wastewater plant.

Spectrophotometry is based on the amount of light that passes through the samples at certain wavelengths, which depends on the physical-chemical characteristics of the samples.

In treated water, the concentrations of organic and inorganic matter are very low, which means that all the wavelengths of the visible spectrum can pass through more easily, giving rise to a more horizontal emission spectrum, without significant changes. It is this absence of variations in the spectral response that makes it difficult to find patterns that allow the pollutant load to be estimated from spectrophotometric data.

The tests carried out showed that the greater is the pollutant load, the easier it is to find correlations between transmittance/absorbance data and the pollutant concentration measured in the treatment plants.

Taking into account how transmittance data evolve with respect to the pollution concentration, it is essential to find the correlations between them. Section 3.1.1 and Section 3.1.2 show an example of the spectrophotometric response of the samples, together with their respective pollutant parameters, at each of the main analysis points in the wastewater treatment plant, in order to show how the pollutant load affects the transmittance and absorbance results.

#### 3.1.1. Wastewater (Raw Water)

**Figure 2:**
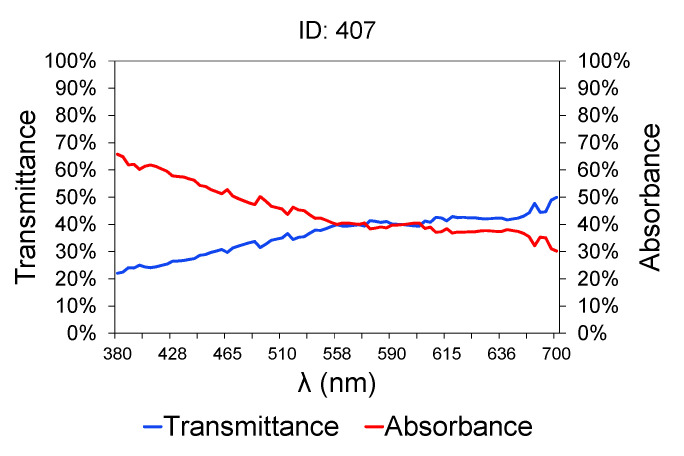
Spectrophotometric data for wastewater sample (raw water).

Figure 2 shows a sample of wastewater taken at the intake of the treatment plant (raw water). Table 2 contains the characteristics of the sample shown in Figure 2. The lower is the transmittance graph, the higher is the contaminant load of the samples, because of their higher turbidity [64,65]. Between 380 and 700 nm, the graph shows an upward slope, which is much steeper between 380 and 558 nm, and then tends to level out [66]. The transmittance graph from 558 nm upwards is typically constant in all the samples, and the transmittance value did not exceed 50% in any case. Therefore, attention should be paid to the region between 380 and 558 nm. A small variation in the transmittance value at 380 nm between different samples involves a large variation in COD, BOD_5_, TSS and TN values [67,68]. Others such as conductivity [69] and PH [70] bear no relation to variations in transmittance and absorbance.

This variation in slope is due to the greater sensitivity of organic matter to ultraviolet light. At low wavelengths in the UV near-visible range, close to 380 nm, organic matter absorbs more radiation and therefore less light is able to pass through the sample (lower transmittance). As the wavelengths are moved away from the ultraviolet/blue area, the organic matter absorbs less light and the change in transmittance is less significant.

#### 3.1.2. Treated Water

**Figure 3:**
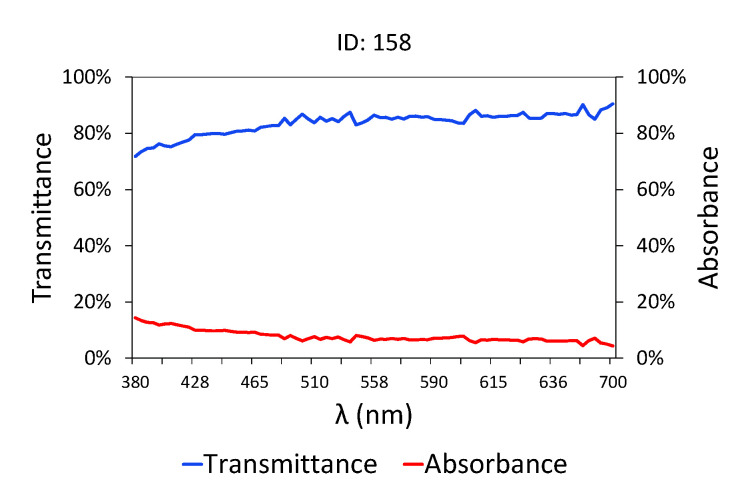
Spectrophotometric data and pollutant parameters for treated samples.

In the case of treated water (Figure 3 and Table 3), that is, effluent water obtained at the treatment plant outlet, the transmittance values are much higher than those shown in Figure 2, as the pollutant load is low in terms of COD, BOD_5_ [71] and TSS. The water at the outlet of the treatment plant has a very high level of transmittance, close to 90% between 445 and 700 nm, where it behaves horizontally, unlike raw water (Figure 2) where the transmittance values seemed to stabilize from 558 nm. Furthermore, the changes in the slope of the graph are only evident in the area close to ultraviolet/blue, given that this is where organic matter is most sensitive.

### 3.2. Regression Models

To find the relations between the contaminating parameters and the spectrophotometric data [8,17], two different approaches have been proposed: one based on MLR analysis [72] and the other by calculating GA [73]. To simplify the equations shown, the following nomenclature is used: *T* is transmittance, *A* is absorbance and the sub-index indicates the wavelength used for its calculation, for instance *T_380_* details that this is the transmittance value measured at 380 nm.

It is necessary to emphasize that the coefficients of the different models presented are specific for the device and the wastewater samples used for its calculation. Therefore, these coefficients should be adjusted to the characteristics of the equipment and the peculiarities of the water in the area where the analysis is carried out.

#### 3.2.1. Multivariate Linear Regressions

Multivariate linear regression models [74,75,76] provide correlations from a set of input variables. However, this method is only valid for datasets that follow a normal distribution [74] (or can be transformed into one). Initially, the tests focused on finding such expressions for a dataset composed of both raw and treated water samples. However, the degree of variability between the two subsets of data composed of both raw and treated water samples was so high that the resulting datasets did not follow a normal distribution, nor was normalization possible despite eliminating outliers.

To illustrate this point more clearly, Figure S1 presents histograms of combined raw and treated water samples for all of the pollutant parameters under study, where each of the histograms shown contains two differentiated zones: one zone on the right that has an approximately normal distribution, which corresponds to the raw water data, and a dominant class (or classes) in terms of frequency in the left region, which corresponds to the treated water samples. This is especially visible in Figure S1a–c,f. Therefore, the combination of raw and treated water data cannot be used for the development of MLR models since they do not follow a normal distribution.

The studies carried out showed that it is only possible to apply this type of model achieving an acceptable minimum degree of adjustment when calculating COD, BOD_5_ and TSS corresponding to raw water. The rest of the parameters, namely P, TN and NO_3_^−^N, cannot be calculated using that method, since, in most cases, either the data could not be standardized or the resulting model had a low level of correlation (lower 50%). The different multivariate linear regression models obtained for the calculation of COD, BOD_5_ and TSS for wastewater (raw water) are shown in the following subsections. The results of the normality tests using the Kolmogorov–Smirnov and Shapiro–Wilk tests as well as the atypical ones detected are shown in Table S1.

##### Chemical Oxygen Demand (COD)

The multivariate linear regression model for calculating COD is shown in Equation (3). This model provided a goodness of fit of 77.4% for the training data. The number of samples used for the calculation of the MLR model was 101, out of a total of 108 samples, after eliminating outliers. From this, 69 samples were used in developing the model, while the remaining samples were used for testing it.

(3) $$COD_{\left(mg/l\right)}=c_{0}-c_{1}\cdot T_{380}+c_{2}\cdot T_{580}-c_{3}\cdot T_{555}+c_{4}\cdot T_{521}\;\bar{R^{2}}=77.4\%$$

*c*_0_ = 844.247657
*c*_1_ = 1752.845
*c*_2_ = 5665.418
*c*_3_ = 7189.785
*c*_4_ = 8046.775

**Figure 4:**
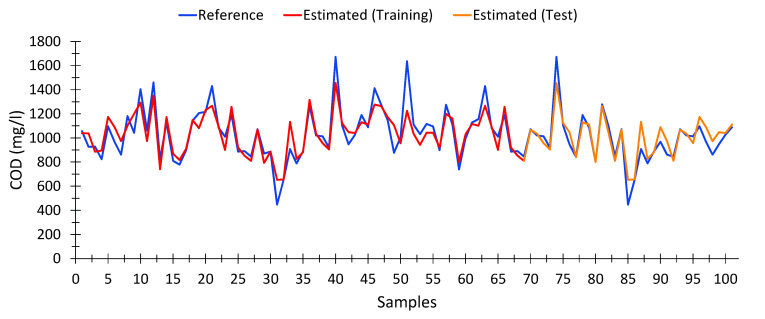
Comparison between COD values measured by the wastewater treatment plant and the values calculated from spectrophotometric data by multivariate linear regression model, for waste water (raw water).

Figure 4 shows a comparison between the COD values obtained at the wastewater treatment plant (blue), the COD values provided by the model shown in Equation (3), both using the training data (red), i.e., the dataset used for building the model up, and the testing dataset (yellow), which is the data that were been used for developing the model.

In general terms, the calculated values are quite close to the expected data.

##### Biological Oxygen Demand at 5 Days (BOD_5_)

The multivariate linear regression model for calculating BOD_5_ is shown in Equation (2). This model provided a goodness of fit of 61.9% for the training data. The model has a low adjustment compared to the previous model. The number of samples used for the calculation of the model was 86, out of a total of 108 samples, after eliminating outliers, so that 70 samples were used in developing the model, while the remaining samples were used for testing it.

(4) $$BOD_{5}{}_{\left(mg/l\right)}=c_{0}-c_{1}\cdot A_{515}+c_{2}\cdot A_{425}-c_{3}\cdot T_{555}\;\bar{R^{2}}=61.9\%$$

*c*_0_ = 2171.855
*c*_1_ = 7898.15
*c*_2_ = 4755.737
*c*_3_ = 2906.184

Figure S2 shows a comparison between the BOD_5_ values provided by the model in Equation (4) (red, the dataset used for building the model up, and yellow, the testing dataset) and the values obtained at the wastewater treatment plant (blue).

In general terms, the calculated values present an appreciable scatter when compared with the reference data.

##### Total Suspended Solids (TSS)

The multivariate linear regression model for calculating TSS is shown in Equation (5). This model provided a goodness of fit of 72.2% for the training data. The number of samples used for the calculation of the MLR model was 92, out of a total of 108 samples, after eliminating outliers, so that 69 samples were used in developing the model, while the remaining samples were used for testing it.

(5) $$TSS_{\left(mg/l\right)}=c_{0}-c_{1}\cdot T_{380}-c_{2}\cdot A_{425}-c_{3}\cdot A_{656}\;\bar{R^{2}}=72.2\%$$

*c*_0_ = 2428.586
*c*_1_ = 5060.755
*c*_2_ = 2928.048

Figure S3 shows a comparison between the TSS values provided by the model in Equation (5) (red, the dataset used for building the model up, and yellow, the testing dataset) and the values obtained at the wastewater treatment plant (blue). As can be seen, in general terms, the model fits the expected TSS values quite well.

As was already observed in the MLR of the COD, the calculated values are quite close to the expected data.

To show the relationship between each of the variables used in the respective models (Equations (3)–(5)) with respect to the pollutant parameter under study, the Supplementary Materials include scatter diagrams for COD (Figure S4), BOD5 (Figure S5) and TSS (Figure S6).

#### 3.2.2. Genetic Algorithms

The MLR models, as shown in the previous section, might be suitable to quantify the pollution load influent to the WWTP, i.e., raw wastewater, in terms of COD and TSS. In contrast, MLR models have difficulties in modeling the behavior of samples with low COD and BOD_5_ levels, i.e., COD lower than 55 mg/L and BOD_5_ lower than 15 mg/L, which is the WWTP effluent. This is due to the fact that the transmittance/absorbance fluctuations in the UV–near visible spectrum are less significant than those observed in the wastewater. This is observed in Figure 3, where the transmittance graph resembles an almost horizontal line.

We aimed to develop a model that could be applied to both raw and treated water, which would overcome the limitations of MLR models and have a good level of accuracy in the estimates. For that reason, we developed a genetic algorithm, more specifically symbolic regression models. For each of the calculated models, 66% of the samples were used as the training data and the remaining 34% were used to validate the data. It is necessary to highlight that the data used for training and testing were selected randomly.

The following subsections show the results obtained by the algorithms, for each of the parameters analyzed: COD, BOD_5_, TSS, P, TN and NO_3_^−^N. Each of them is followed by its correlation formula, as well as a comparison with the expected values of the polluting parameters.

##### Chemical Oxygen Demand (COD)

The model for calculating COD from spectrophotometric data is shown in Equation (6). This model presented an average Pearson goodness-of-fit for nonlinear regressions of 90.95%, with a similar adjustment in the training data (95.07%) and the test data (90.93%). In total, 188 samples out of 196, taken from different treatment plants and days, as well as input water (raw water) and output water (treated water), were used for the calculation. The optimal model was achieved in the generation number 84 of a maximum of 100.

(6) $$COD_{\left(mg/l\right)}=\left[\left(\left(c_{0}\cdot A_{594}-c_{1}\cdot A_{557}\right)+\left(c_{2}\cdot A_{380}-c_{3}\cdot A_{521}\right)\right)+\left(\left(c_{4}\cdot A_{425}-c_{5}\cdot A_{575}\right)+\left(c_{6}\cdot A_{445}-c_{7}\cdot A_{520}\right)\right)\right]\cdot c_{8}+c_{9}$$

*c*_0_ = 2.4268
*c*_1_ = 2.7910
*c*_2_ = 2.5317
*c*_3_ = 2.6341
*c*_4_ = 2.3278
*c*_5_ = 2.6879
*c*_6_ = 2.4569
*c*_7_ = 2.7717
*c*_8_ = 1191.8
*c*_9_ = −263.45

This model is based on eight wavelengths for its calculation, namely 380, 425, 445, 520, 521, 570, 575 and 594 nm, more specifically from the absorbance data. However, not all variables (wavelengths) are equally relevant. As shown in Table 4, 380, 425, 445 and 594 nm are the most relevant variables, with an impact factor close to 17%, while the remaining variables are at around 5%.

It is important to note that the wavelengths with the highest impact index were those belonging to the violet zone of the visible spectrum (380–450 nm), which was to be expected, since organic matter is far more sensitive to those wavelengths. Likewise, it was also observed that the wavelengths close to red showed a greater interaction with the water samples. This suggests that the use of near-infrared wavelengths would provide better characterization of the samples.

**Figure 5:**
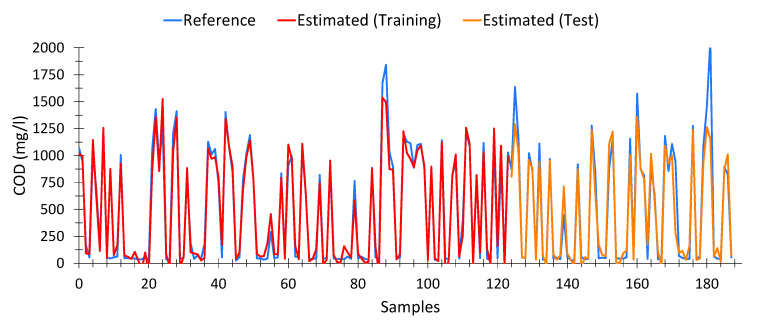
Comparison between COD values measured by the wastewater treatment plant and the values calculated from spectrophotometric data, according to Equation (6).

As shown in Figure 5, the estimates provided by the model (both for training (red) and test (orange) data) fit precisely with the expected results (Blue). We can see that for very high values of COD (>1600 mg/L) the estimates tend to be lower than expected. However, the results could be adequate to provide an early warning system. However, at low values of COD [77], the model is able to provide a fairly certain estimation from spectrophotometric data, which was not possible with linear models.

Table A1 in Appendix B shows 15 random records, which include the absorbance values obtained for each of the variables used in the model (Equation (6)), as well as the expected COD values (Reference) and those calculated by the model (Estimated).

It can be seen that the results calculated are very similar to those expected, even when the COD level is low. The model obtained by means of the genetic algorithm was able to precisely estimate COD values from the data provided by the spectrophotometer.

##### Biological Oxygen Demand at 5 Days (BOD_5_)

To calculate the model for BOD_5_, 162 samples were used out of a total of 196 samples after eliminating outliers—a lower number than before—due to two aspects: the existence of outliers and measurements where BOD_5_ data were not available. The calculated model is shown in Equation (7). This model showed an average Pearson goodness-of-fit of 90.71% (training data) and 90% for test data (88.23% average). In addition, the model is valid for water samples with high levels of pollution (raw water) as well as with low levels of pollution (treated water). The optimal model was achieved in the 98th generation.

(7) $$BOD_{5}{}_{\left(mg/l\right)}=\left[\frac{c_{0}\cdot A_{574}\cdot c_{1}\cdot T_{585}}{c_{2}\cdot A_{655}-c_{3}\cdot T_{415}}\cdot \frac{c_{4}\cdot T_{585}\cdot c_{5}\cdot A_{445}}{c_{6}\cdot A_{655}-c_{7}\cdot T_{415}}\right]\cdot c_{8}+c_{9}$$

*c*_0_ = 2.0733
*c*_1_ = 1.3974
*c*_2_ = −1.0226
*c*_3_ = 1.2453
*c*_4_ = 1.3974
*c*_5_ = −0.1356
*c*_6_ = −1.0226
*c*_7_ = 1.2453
*c*_8_ = −10078
*c*_9_ = −18.784

This model is based on five wavelengths for its calculation: 415, 445, 574, 585 and 655 nm. However, not all variables (wavelengths) are equally relevant. As can be seen in Table 5, the most relevant wavelengths are those closest to the violet area [78], although the wavelengths close to red have a similar level of importance, although in smaller proportions.

**Figure 6:**
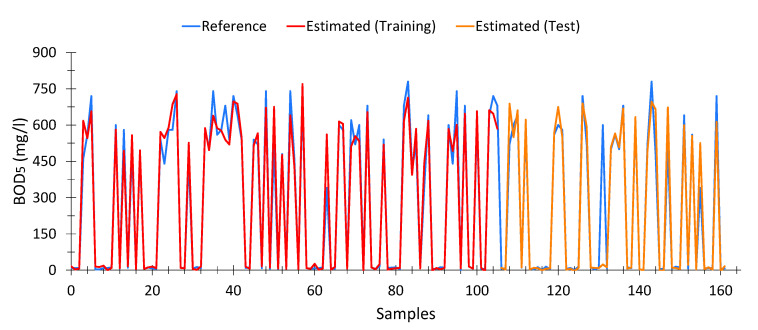
Comparison between BOD_5_ values measured by the wastewater treatment plant and the values calculated from spectrophotometric data, according to Equation (7).

Figure 6 shows the estimations of the genetic algorithm. As can be seen, the adjustment is acceptable, although in general terms the results seem to be a little lower than expected, but this fluctuation is not significant.

Table A2 (Appendix B) shows 15 records taken at random, where the results obtained by the model are very similar to those that were expected. Each record contains the spectrophotometric data as well as the expected (reference) values calculated by the model.

##### Total Suspended Solids (TSS)

Equation (8) shows the model calculated for total suspended solids. This model presented an average Pearson goodness-of-fit of 87.47% (94.67% with the training data and 90% with the test data). In total, 172 samples were used out of the 196 samples, after eliminating outliers.

(8) $$TSS_{\left(mg/l\right)}=\frac{c_{0}\cdot A_{574}+c_{1}\cdot T_{558}}{c_{2}\cdot T_{485}}\cdot \left(\frac{c_{3}\cdot T_{565}}{c_{4}\cdot T_{380}}+c_{5}\cdot T_{632}\right)\cdot c_{6}+c_{7}$$

*c*_0_ = −0.062545
*c*_1_ = 2.6249
*c*_2_ = 3.4131
*c*_3_ = 2.5468
*c*_4_ = 3.3361
*c*_5_ = 0.46423
*c*_6_ = 782.89
*c*_7_ = −779.73

The model makes use of six variables (wavelengths): transmittance at 380, 485, 558, 565 and 632 nm and absorbance at 574 nm. However, the most relevant are 380 and 485 nm, as shown in Table 6. Likewise, we observe that, as wavelengths approach the infrared spectrum, the relative weight of these variables decreases significantly, as is the case with 632 nm.

This shows that particles in suspension are far more sensitive to wavelengths close to violet than to other wavelengths.

**Figure 7:**
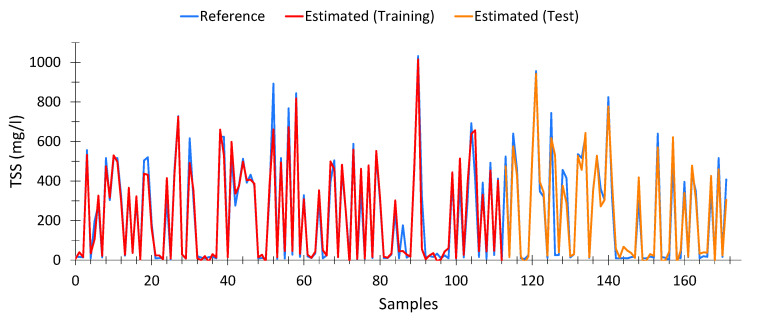
Comparison between TSS values measured by the wastewater treatment plant and the values calculated from spectrophotometric data, according to Equation (8).

Figure 7 shows the results obtained with the calculated model. As can be seen, the fit is adequate, even at high TSS levels.

Similar to the previous cases, Table A3 (Appendix B) shows 15 cases chosen at random, in order to verify the good performance of the model, even at low levels of TSS.

##### Phosphorus (P)

The model calculated for P is shown in Equation (9). The model presented an average Pearson goodness-of-fit of 74.01% (74.28% with the training data and 78.33% with the test data), with the optimum being raised at generation 38 of a maximum of 100. In total, 175 data were used for its calculation.

(9) $$P_{\left(mg/l\right)}=\left[\left(\frac{c_{0}\cdot T_{430}}{c_{1}\cdot T_{585}}-c_{2}\cdot T_{650}\right)\cdot \left(c_{3}\cdot T_{425}-c_{4}\cdot T_{585}\right)\;\cdot \left(c_{5}\cdot T_{450}-c_{6}\cdot T_{650}\right)\cdot c_{7}+c_{8}\right]$$

*c*_0_ = 1.53
*c*_1_ = 0.8773
*c*_2_ = 1.1618
*c*_3_ = 1.5294
*c*_4_ = 0.8773
*c*_5_ = 2.2034
*c*_6_ = 1.1618
*c*_7_ = −40.766
*c*_8_ = 9.0573

The calculated model uses five wavelengths: 425, 430, 450, 585 and 650 nm. Once again, the most representative wavelengths were those closest to the violet zone [79], as shown in Table 7. As with the total suspended solids model, the weight of the wavelengths decreases as it approaches the infrared portion of the spectrum.

The model generated to estimate phosphorus levels from spectrophotometric data has a lower adjustment compared to the previous models and presents systematic inaccuracies for higher concentrations. The calculated model was only able to accurately estimate *p* values lower than or equal to 9 mg/L. This characteristic can be seen in Figure S7, where the estimated values are never higher than that value. Table A4 (Appendix B) shows 15 cases chosen at random, in order to verify the performance of the model.

##### Total Nitrogen (TN)

The model for Total Nitrogen (TN) is shown in Equation (10). This model had an average Pearson goodness-of-fit of 79.93% (85.91% with the training data and 85.91% with the test data), having been calculated from 175 samples out of the total of 196 after eliminating outliers. The optimum was raised at generation 87.

(10) $$TN_{\left(mg/l\right)}=\left[\left(c_{0}\cdot T_{655}-c_{1}\cdot T_{585}\right)\cdot \left(c_{2}\cdot T_{640}-c_{3}\cdot T_{510}\right)\cdot \left(c_{4}\cdot T_{557}-c_{5}\cdot T_{585}\right)\;\cdot \left(c_{6}\cdot T_{640}-c_{7}\cdot T_{500}\right)\cdot c_{8}+c_{9}\right]$$

*c*_0_ = 1.3315
*c*_1_ = 0.85214
*c*_2_ = 2.1725
*c*_3_ = 1.6762
*c*_4_ = 1.4023
*c*_5_ = 0.85214
*c*_6_ = 2.1725
*c*_7_ = 1.6605
*c*_8_ = −1271.6
*c*_9_ = 83.172

The model makes use of six wavelengths: 500, 510, 557, 585, 640 and 655 nm. As shown in Table 8, the most representative wavelengths used to calculate the nitrogen content of water were those closest to infrared. This has already been highlighted in [80], where nitrogen has a higher correlation with wavelengths close to the infrared spectrum [81].

Figure S8 shows the results provided by the model described in 10. As can be seen, the formula works well within a certain range of nitrogen values between 20 and 75 mg/L, but worsens slightly outside that range, albeit not significantly.

Within that range, the results provided by the model were very close to the reference values, as shown in Table A5 (Appendix B).

##### Nitrate Nitrogen (NO_3_^−^N)

Nitrogen nitrate in water can be calculated from Equation (11). The model presented an average Pearson goodness-of-fit of 81.26% (81.26% with the training data and 83.46% with the test data). In total, 175 samples were used for calculation out of 196 samples after eliminating outliers. The optimum was raised at generation 81 of 100.

(11) $$NO_{3^{-}}N_{\left(mg/l\right)}=\left(\frac{c_{0}\cdot A_{560}\cdot c_{1}\cdot A_{560}}{c_{2}\cdot A_{607}\cdot c_{3}\cdot A_{624}}\cdot \frac{c_{4}\cdot A_{428}\cdot c_{5}\cdot T_{385}}{c_{6}\cdot A_{607}\cdot c_{7}\cdot A_{645}}\cdot c_{8}+c_{9}\right)$$

*c*_0_ = 2.2576
*c*_1_ = 2.2576
*c*_2_ = −0.53193
*c*_3_ = 1.5017
*c*_4_ = 0.66989
*c*_5_ = 2.277
*c*_6_ = −0.53193
*c*_7_ = −0.50608
*c*_8_ = −0.010536
*c*_9_ = −0.12637

The model uses the following six wavelengths: 385, 428, 560, 607, 624 and 645 nm. The tests showed that NO_3_^−^N has a higher correlation with wavelengths close to 600 nm, as shown in Table 9.

Figure S9 shows the results obtained for different water samples. Considering that the vertical scale in the figure is shown in 2 mg/L intervals, the discrepancies between the calculated values and the reference values are not significant.

Table A6 (Appendix B) shows 15 cases chosen at random, where the high degree of similarity between the data provided by the model and the values calculated in the wastewater treatment plants can be observed.

#### 3.2.3. Decision Support System Proposal

To carry out an in-depth analysis of the different models calculated, the RMSE and Error Rate E(%) were calculated following Equations (1) and (2), respectively. The results are shown in Table 10.

As shown in Table 10, the COD error value provided by the MLR model (−0.096%) is lower than that obtained by the genetic algorithm (−2.374%). Nevertheless, we must take into account that a different number of samples was used; GA takes treated water into account; thus, although it is true that the MLR model showed better performance than that provided by the genetic algorithm, its use is limited to raw water.

Thus, if the samples which we seek to obtain the COD value for are only samples of raw water, then the MLR model presents the best results. However, if we want to carry out the study on both types of water (raw and treated), then the genetic algorithm must be used to calculate the COD. The genetic algorithm presented the best performance for the remaining parameters.

Looking into the contribution of the wavelengths to the statistical models, not all wavelengths have the same weight in the models, as shown in Table 4, Table 5, Table 6, Table 7, Table 8 and Table 9. In general terms, those wavelengths closer to violet have a greater weight, which is understandable considering that organic matter reacts more to UV than to other wavelengths. On the other hand, wavelengths close to IR also have a greater importance in the calculation of inorganic parameters such as TN.

In addition, although in general terms the models calculated by means of the genetic algorithms present a better performance, it is necessary to emphasize that these models make use of a greater number of variables (wavelengths) than the models of linear regression. This implies a greater time of analysis and an increase of the load of the system as well as the price of the equipment since more LEDs is necessary. Therefore, the choice of the model will depend on the application.

Table 11 shows the different wavelengths used for the calculation of the six pollutant parameters, where each cell shows the degree of importance of that wavelength in its calculation, accompanied by a color code, for greater clarity of the reader: green (high relevance), blue (medium-high relevance), orange (medium-low relevance) and red (low relevance). The coefficients shown were determined automatically by the SPSS software and gpLearn from the *P*-value of the variables introduced in the different models.

As shown in Table 11, the contaminating parameters related to organic matter, such as COD and BOD5, show a greater interaction with wavelengths close to violet and with a lower extent with wavelengths in the order of 500–550 nm (green).

On the other hand, the parameters more related to inorganic matter such as total nitrogen (TN) are more sensitive to wavelengths close to the infrared (IR); in fact, the TN is calculated using NIRS techniques (near infrareds) [82,83].

At this point, it is necessary to make a comparison between the wavelengths present in the MLR and GA models. As shown in Table 11, the wavelengths selected by both methodologies are similar, especially in the ultraviolet zone, where, for example, the wavelength of 380 nm is present for both COD and BOD_5_ in both types of models (MLR and GA). On the other hand, it is necessary to take into account that GA models are valid for both raw and treated water and therefore it is logical to think that the number of wavelengths used is greater than that required to model only raw water. Despite this, there are similarities between both types of models.

## 4. Conclusions

In this paper, we show different models that enable us to estimate the concentration of COD, BOD_5_, TSS, P, TN and NO_3_^−^N from the absorbance and transmittance measures of the water samples, within the range of 380–700 nm. These models can be used to estimate the pollutant load of both the incoming water (raw water) and the outgoing water (treated water), without the need for any pre-treatment or chemicals.

The research focused on two types of models: multivariate linear regression and genetic algorithm. The tests carried out determined that the models calculated by means of genetic algorithms are able to obtain valid estimates principally for five of the pollutants under study (COD, BOD_5_, TSS, TN and NO_3_^−^N), including both raw and treated waste water in the adjustments, with an error rate below 4% in all the models. In the case of the MLR models, their adequacy is limited to COD and TSS, while BOD_5_ presents a poor fit. In contrast to GA, the MLR models presented better error rates than those calculated by genetic algorithms, with an error rate of less than 0.5% for COD and TSS. However, MLR models are limited to raw water samples. The variability of wastewater samples makes it difficult for MLR models to find a single valid model for both influent (raw water) and effluent (treated) wastewater. However, models calculated by means of genetic algorithms have proven to be reliable enough to find common patterns among the different types of samples, in order to achieve a valid calculation model for all types of wastewater (raw and treated).

The current research also provides a clearer view of the effect that each of the UV–near visible and visible wavelengths (380–700 nm) have on the estimation of each of the polluting parameters. As shown in Table 11, the wavelengths having the greatest effect on the calculation are those corresponding to the UV–near visible (380–400 nm) and near-infrared (600–700 nm) zones, with a relevance (impact) of 17–20% in the model calculation, while the zone between 500 and 600 nm is the least relevant, with an impact of around 5%, albeit with some exceptions, such as TSS (around 10%).

COD, BOD_5_, TSS and P depend mainly on the UV zone for their calculation, representing (in the case of models calculated with the GA) around 52%, 40%, 70% and 40%, respectively. On the other hand, TN and NO_3_^−^N depend mainly on the IR zone.

In this research work, a completely different approach was sought to what is followed by systems such as those described in [59,60,61,62], which base their operation on the analysis of the turbidity of wastewater samples, that is, on a turbidimeter. A turbidimeter analyzes the samples at a single wavelength (typically belonging to the infrared spectrum). In contrast, the system developed and described in this manuscript makes use of 81 different wavelengths. This allows a much more precise knowledge of the physical-chemical and bacteriological properties of the samples, since wavelengths close to UV are of great importance to know the behavior of organic matter, while wavelengths close to red (or infrared) enable analyzing the behavior of inorganic matter with high precision. Therefore, the use of multiple wavelengths makes it possible to obtain adequate estimates of the pollution load of wastewater.

This research can serve as a starting position for future continuous real-time monitoring of the whole sanitation system that includes the deployment of simpler, smaller and more cost-effective equipment for the study of the pollutant load in sewage networks, capable of obtaining valuable information from the spectrophotometry-based statistical models and providing early warning. This distribution of this equipment along the networks can be especially useful during rain episodes, when the pollution load of sanitation networks tends to rise, and represents a danger to the environment. Therefore, having rapid information on this type of parameters is essential for preventing and reducing environmental disasters.

## Figures and Tables

**Table 1:** Pollutant parameters supported by the equipment.

Variable	Description	Test Standard/Procedure
DBO5	Respirometric method	SM 5210 D
COD	Dichromate method with UV–VIS spectroscopy	ISO 6060:1989
TSS	Settleable solids	SM 2540 F
NO_3_^−^N	Dimethylphenol spectrometric method	ISO 7890-1
TN	Persulfate digestion with UV–VIS spectroscopy	SM 4500–NC
TP	Ascorbic Acid Method complemented with UV–VIS spectroscopy	SM 4500-P B

**Table 2:** Pollutant parameters for wastewater sample (raw water).

Polluting Parameters	Value
COD	763 mg/L
BOD5	500 mg/L
TSS	304 mg/L
Phosphorus (P)	9.1 mg/L
Total Nitrogen (TN)	74 mg/L
NO_3_^−^N	0.5 mg/L
PH	7.59
Conductivity	2770 µS/cm

**Table 3:** Pollutant parameters for wastewater sample (raw water).

Polluting Parameters	Value
COD	52 mg/L
BOD5	9 mg/L
TSS	14 mg/L
Phosphorus (P)	2.5 mg/L
Total Nitrogen (TN)	16.6 mg/L
NO_3_^−^N	10.3 mg/L
PH	7.56
Conductivity	2580 µS/cm

**Table 4:** Impact indices of model variables genetic algorithm COD.

Variable	Impact
A380 nm	17.021%
A425 nm	17.652%
A445 nm	17.691%
A520 nm	5.670%
A521 nm	5.417%
A570 nm	5.067%
A575 nm	4.966%
A594 nm	17.015%

**Table 5:** Impact indices of model variables genetic algorithm BOD_5_.

Variable	Impact
T415 nm	21.211%
A445 nm	18.522%
A574 nm	18.030%
T585 nm	13.924%
A655 nm	15.312%

**Table 6:** Impact indices of model variables genetic algorithm TSS.

Variable	Impact
T380 nm	34.059%
T485 nm	34.975%
A574 nm	0.018%
T558 nm	11.166%
T565 nm	10.600%
T632 nm	1.552%

**Table 7:** Impact indices of model variables genetic algorithm P.

Variable	Impact
T425 nm	19.057%
T430 nm	19.035%
T450 nm	18.130%
T585 nm	12.355%
T650 nm	7.024%

**Table 8:** Impact indices of model variables genetic algorithm TN.

Variable	Impact
T500 nm	7.254%
T510 nm	6.639%
T557 nm	19.502%
T585 nm	9.426%
T640 nm	18.765%
T655 nm	19.714%

**Table 9:** Impact indices of model variables genetic algorithm NO_3_^−^N.

Variable	Impact
T385nm	8.257%
A428nm	14.054%
A560nm	16.613%
A607nm	20.243%
A624nm	11.210%
A645nm	11.924%

**Table 10:** Root-mean-square deviation (RMSD) and index error (Er) models.

Model	Number Samples	Parameter	RMSD	Er (%)
Multivariate Linear Regression (Raw water only)	101	COD	95,910	−0.096%
	86	BOD_5_	134,372	−5.540%
	92	TSS	62,197	0.295%
Genetic Algorithm (Raw and treated water)	188	COD	137,048	−2.374%
	162	BOD_5_	69,051	−0.173%
	172	TSS	67,159	0.621%
	175	P	2037	−2.634%
	175	TN	11,783	0.147%
	175	NO_3_^−^N	2323	−3.928%

**Table 11:** Summary of wavelength relevance in the statistical models to characterize pollutant parameters calculated through GA.

	Multivariate Linear Regression			Genetic Algorithms					
	COD	BOD_5_	TSS	COD	BOD_5_	TSS	P	TN	NO_3_^−^N
380 nm	32.834%		30.291%	17.021%		34.059%			
385 nm									8.257%
415 nm					21.211%				
425 nm		26.488%	29.334%	17.652%			19.057%		
428 nm									14.054%
430 nm							19.035%		
445 nm				17.691%	18.522%				
450 nm							18.130%		
485 nm						34.975%			
500 nm								7.254%	
510 nm								6.639%	
515 nm		18.780%							
520 nm				5.670%					
521 nm	14.794%			5.417%					
555 nm	14.207%	12.411%							
557 nm								19.502%	
558 nm						11.166%		9.426%	
560 nm									16.613%
565 nm						10.600%			
570 nm				5.067%					
574 nm					18.030%	≈0%			
575 nm				4.966%					
580nm	13.283%								
585 nm					13.924%		12.355%		
594 nm				17.015%					
607 nm									20.243%
624 nm									11.210%
632 nm						1.552%			
640 nm								18.765%	
645 nm									11.924%
650 nm							7.024%		
655 nm					15.312%			19.714%	
656 nm			11.875%						

## Supplementary Materials

The following are available online at https://www.mdpi.com/1424-8220/20/19/5631/s1, Figure S1: Histogram of combined raw and treated water samples. (A) COD, (B) BOD5, (C) TSS, (D) P, (E) TN and (F) NO_3_^−^N, Table S1: Basic characteristics of raw water data sets for MLR study, Figure S2: Comparison between BOD5 values measured by the wastewater treatment plant and the values calculated from spectrophotometric data by multivariate linear regression model, for waste water (raw water), Figure S3: Comparison between TSS values measured by the wastewater treatment plant and the values calculated from spectrophotometric data by multivariate linear regression model, for waste water (raw water), Figure S4: Scatter plot of the variables used in the MLR model for calculating COD with respect to: (A) Transmittance at 380 nm, (B) Transmittance at 521 nm, (C) Transmittance at 555 nm and (D) Transmittance at 580 nm, Figure S5: Scatter plot of the variables used in the MLR model for calculating BOD5 with respect to: (A) Transmittance at 555 nm, (B) Absorbance at 425 nm and (C) Absorbance at 515 nm, Figure S6: Scatter plot of the variables used in the MLR model for calculating TSS with respect to: (A) Transmittance at 380 nm, (B) Absorbance at 425 nm and (C) Absorbance at 655 nm, Figure S7: Comparison between P values measured by the wastewater treatment plant and the values calculated from spectrophotometric data, according to Equation (9), Figure S8: Comparison between TN values measured by the wastewater treatment plant and the values calculated from spectrophotometric data, according to Equation (10), Figure S9: Comparison between NO_3_^−^N values measured by the wastewater treatment plant and the values calculated from spectrophotometric data, according to Equation (11).

## Appendix B

**Table A1:** Example cases for COD estimation.

Abs. λ380 nm	Abs. λ425 nm	Abs. λ445 nm	Abs. λ520 nm	Abs. λ521 nm	Abs. λ570 nm	Abs. λ575 nm	Abs. λ594 nm	COD	
								Reference	Estimated
0.6849	0.6150	0.5584	0.4286	0.4190	0.3686	0.3712	0.3532	1072.000	1019.642
0.6595	0.5925	0.5068	0.4089	0.3997	0.3387	0.3409	0.3317	947.000	989.649
0.1468	0.1029	0.0970	0.0712	0.0664	0.0646	0.0645	0.0693	54.000	84.371
0.6948	0.6092	0.5478	0.4206	0.4112	0.3523	0.3548	0.3478	1042.000	1143.886
0.6514	0.5852	0.5439	0.4505	0.4405	0.3854	0.3882	0.3758	672.000	608.425
0.2238	0.1776	0.1539	0.1310	0.1256	0.1138	0.1139	0.1141	167.000	114.573
0.7042	0.6147	0.5676	0.4148	0.4055	0.3554	0.3579	0.3456	1192.000	1256.427
0.1438	0.1103	0.0973	0.0742	0.0693	0.0704	0.0702	0.0723	52.000	48.711
0.7268	0.6568	0.6107	0.4691	0.4587	0.4033	0.4063	0.3914	1206.000	1039.073
0.8657	0.7763	0.7350	0.5606	0.5481	0.4599	0.4638	0.4446	1412.000	1352.363
0.1487	0.1088	0.0945	0.0753	0.0704	0.0674	0.0672	0.0699	53.000	56.831
0.1556	0.1181	0.0992	0.0794	0.0745	0.0804	0.0804	0.0842	50.000	46.730
0.1363	0.0949	0.0807	0.0639	0.0591	0.0628	0.0626	0.0663	33.000	33.484
0.1504	0.1096	0.0917	0.0741	0.0693	0.0702	0.0701	0.0662	44.000	33.788
0.1525	0.1159	0.0948	0.0770	0.0721	0.0735	0.0734	0.0717	56.000	42.996

**Table A2:** Example cases for BOD_5_ estimation.

Trans. λ415 nm	Trans. λ585 nm	Abs. λ445 nm	Abs. λ574 nm	Abs. λ655 nm	BOD_5_	
					Reference	Estimated
0.76629	0.87322	0.10162	0.06002	0.05524	9.000	6.420
0.24403	0.45556	0.55325	0.34426	0.27607	460.000	618.008
0.27323	0.48592	0.49108	0.31802	0.25519	560.000	546.023
0.22075	0.42956	0.58969	0.36959	0.29248	720.000	656.963
0.20025	0.38834	0.61710	0.43081	0.35095	600.000	581.177
0.75852	0.85732	0.09879	0.06732	0.06093	7.000	7.909
0.30964	0.52375	0.45107	0.29201	0.23394	580.000	493.581
0.74203	0.84762	0.11473	0.07572	0.07354	10.000	15.819
0.28582	0.50567	0.46952	0.30787	0.23439	500.000	558.014
0.77097	0.87022	0.08342	0.06775	0.05189	7.000	4.297
0.28026	0.48173	0.47856	0.32273	0.26617	480.000	495.409
0.76758	0.86484	0.09159	0.06533	0.06259	6.000	5.035
0.74707	0.85266	0.09923	0.07717	0.07499	10.000	11.608
0.74225	0.85333	0.10753	0.07400	0.05520	7.000	14.561
0.75661	0.86687	0.09744	0.06849	0.06876	6.000	8.296

**Table A3:** Example cases for TSS estimation.

Trans. λ380 nm	Trans. λ485 nm	Trans. λ558 nm	Trans. λ565 nm	Trans. λ632 nm	Abs. λ574 nm	TSS	
						Reference	Estimated
0.71805	0.85293	0.86436	0.85679	0.85385	0.06707	14.000	16.465
0.17321	0.31715	0.38169	0.37671	0.42695	0.41352	556.000	532.238
0.71176	0.85818	0.87089	0.86328	0.88086	0.06131	10.000	34.512
0.44954	0.60752	0.64443	0.63804	0.67229	0.19692	196.000	105.120
0.23555	0.37954	0.43810	0.43281	0.47263	0.35013	280.000	326.214
0.70113	0.83559	0.84809	0.84060	0.86102	0.07077	14.000	22.257
0.18672	0.32875	0.39232	0.38728	0.44135	0.39805	516.000	474.119
0.27633	0.44280	0.50279	0.49716	0.54584	0.29201	304.000	317.133
0.20826	0.36028	0.43938	0.43409	0.48646	0.34426	516.000	529.615
0.19314	0.33723	0.40594	0.40083	0.46534	0.36959	516.000	496.820
0.26650	0.42252	0.47833	0.47283	0.53102	0.30422	324.000	295.014
0.66467	0.80578	0.82254	0.81519	0.82097	0.08772	23.000	27.927
0.19669	0.33466	0.38786	0.38285	0.42378	0.39400	348.000	366.015
0.68431	0.85071	0.83658	0.82916	0.87437	0.08630	5.000	6.355
0.72555	0.85706	0.86291	0.85535	0.86743	0.06414	6.000	8.570

**Table A4:** Example cases for P estimation.

Trans. λ425 nm	Trans. λ430 nm	Trans. λ450 nm	Trans. λ585 nm	Trans. λ650 nm	P	
					Reference	Estimated
0.76348	0.78803	0.79807	0.84547	0.86287	0.400	0.879
0.75775	0.77838	0.78847	0.86838	0.86515	2.600	2.443
0.24537	0.26567	0.29388	0.43165	0.48103	8.200	9.064
0.76757	0.79379	0.80362	0.85966	0.87870	0.700	1.494
0.29621	0.33102	0.35410	0.50567	0.54240	11.500	9.028
0.25278	0.27821	0.30349	0.44776	0.49307	7.200	9.070
0.79540	0.81032	0.79494	0.86041	0.86512	1.100	0.103
0.76358	0.83164	0.79458	0.87531	0.86525	1.400	1.136
0.30329	0.33745	0.36199	0.49713	0.54051	10.000	8.951
0.22378	0.24929	0.27953	0.44088	0.47647	11.000	9.106
0.17840	0.19534	0.22203	0.35499	0.38754	10.800	9.088
0.25701	0.28659	0.31936	0.44796	0.48733	11.300	9.057
0.75361	0.79287	0.79741	0.86315	0.86191	0.700	1.743
0.29637	0.32694	0.35853	0.49760	0.55641	8.700	9.008
0.17356	0.18391	0.21443	0.35122	0.41531	9.900	9.050

**Table A5:** Example cases for TN estimation.

Trans. λ500 nm	Trans. λ510 nm	Trans. λ557 nm	Trans. λ585 nm	Trans. λ640 nm	Trans. λ655 nm	TN	
						Reference	Estimated
0.82757	0.80397	0.82113	0.85584	0.86375	0.86940	25.000	22.087
0.44955	0.45871	0.50539	0.52375	0.55827	0.58352	73.000	60.330
0.36769	0.38270	0.43492	0.44776	0.48237	0.51313	66.000	67.600
0.36531	0.37489	0.42788	0.45556	0.48742	0.52958	58.000	66.548
0.86872	0.84108	0.87294	0.86815	0.88365	0.87850	15.400	18.737
0.32128	0.32217	0.37064	0.39510	0.43704	0.48072	65.000	71.143
0.85905	0.83739	0.85368	0.84908	0.87566	0.86752	17.200	21.488
0.43282	0.43977	0.48290	0.49760	0.53349	0.56887	68.000	63.238
0.28615	0.29335	0.33406	0.36080	0.38741	0.43757	92.000	75.947
0.86767	0.84051	0.86005	0.87977	0.89105	0.86902	25.000	21.501
0.85770	0.82966	0.84810	0.85738	0.86480	0.86812	25.000	28.133
0.33833	0.34440	0.38470	0.41056	0.43200	0.48097	66.000	73.640
0.85727	0.82356	0.83882	0.85370	0.86860	0.86295	17.800	26.685
0.27823	0.27440	0.34322	0.34476	0.37727	0.42773	68.000	74.717
0.87407	0.84157	0.89042	0.85226	0.87565	0.87850	17.100	17.848

**Table A6:** Example cases for NO_3_^−^N estimation.

Trans. λ385 nm	Abs. λ428 nm	Abs. λ560 nm	Abs. λ607 nm	Abs. λ624 nm	Abs. λ645 nm	NO_3_^−^N	
						Reference	Estimated
0.7427	0.0939	0.0825	0.0578	0.0595	0.0621	12.000	14.538
0.7051	0.0972	0.0631	0.0563	0.0634	0.0557	10.600	9.169
0.7358	0.0981	0.0582	0.0540	0.0502	0.0562	11.100	11.164
0.1233	0.8244	0.5345	0.4948	0.4640	0.4615	0.300	0.085
0.7273	0.1079	0.0723	0.0685	0.0719	0.0631	10.600	7.199
0.2677	0.4623	0.3142	0.2838	0.2679	0.2599	0.300	0.703
0.2635	0.4870	0.3112	0.2908	0.2743	0.2673	0.400	0.637
0.7500	0.0940	0.0678	0.0610	0.0636	0.0562	11.700	9.151
0.7243	0.0738	0.0653	0.0527	0.0624	0.0580	6.300	8.508
0.2279	0.5592	0.3987	0.3626	0.3448	0.3307	0.300	0.388
0.2560	0.4910	0.3422	0.3139	0.3004	0.2917	0.300	0.523
0.2824	0.4625	0.3037	0.2798	0.2573	0.2484	0.500	0.790
0.7362	0.1000	0.0694	0.0621	0.0601	0.0538	12.200	10.685
0.2062	0.5632	0.3706	0.3378	0.3253	0.3150	0.500	0.393
0.1768	0.6745	0.4240	0.3851	0.3599	0.3498	0.300	0.311

## References

[1] Puertas J., Suárez J., Anta J. (2008). Gestión de las Aguas Pluviales. Implicaciones en el Diseño de los Sistemas de Saneamiento y Drenaje Urbano. Monografía M98.

[2] Ward S., Butler D. (2009). Compliance with the Urban Waste Water Treatment Directive: European Union City Responses in Relation to Combined Sewer Overflow Discharges.

[3] Naves J., Anta J., Suárez J., Puertas J. (2020). Hydraulic, wash-off and sediment transport experiments in a full-scale urban drainage physical model. Sci. Data.

[4] Anta J., Pena E., Suarez J., Cagiao J. (2007). A BMP selection process based on the granulometry of runoff solids in a separate urban catchment. Water SA.

[5] Bourgeois W., Burgess J., Stuetz R. (2001). On-line monitoring of wastewater quality: A review. J. Chem. Technol. Biotechnol..

[6] Melendez-Pastor I., Almendro-Candel M.B., Pedreno J.N., Gómez I., Lillo M.G., Hernández E.I. (2013). Monitoring Urban Wastewaters’ Characteristics by Visible and Short Wave Near-Infrared Spectroscopy. Water.

[7] Brzezińska A., Zawilski M., Sakson G. (2016). Assessment of pollutant load emission from combined sewer overflows based on the online monitoring. Environ. Monit. Assess..

[8] Chen B., Wu H., Li S.F.Y. (2014). Development of variable pathlength UV–vis spectroscopy combined with partial-least-squares regression for wastewater chemical oxygen demand (COD) monitoring. Talanta.

[9] Gondal M.A., Hussain T. (2017). Determination of poisonous metals in wastewater collected from paint manufacturing plant using laser-induced breakdown spectroscopy. Talanta.

[10] Ferree M., Shannon R.D. (2001). Evaluation of a second derivative UV/visible spectroscopy technique for nitrate and total nitrogen analysis of wastewater samples. Water Res..

[11] Qin X., Gao F., Chen G. (2012). Wastewater quality monitoring system using sensor fusion and machine learning techniques. Water Res..

[12] Lepot M., Torres A., Höfer T., Caradot N., Gruber G., Aubin J.-B., Bertrand-Krajewski J.-L. (2016). Calibration of UV/Vis spectrophotometers: A review and comparison of different methods to estimate TSS and total and dissolved COD concentrations in sewers, WWTPs and rivers. Water Res..

[13] Wolf C., Gaida D., Stuhlsatz A., Ludwig T., McLoone S.F., Bongards M. (2011). Predicting organic acid concentration from UV/vis spectrometry measurements—A comparison of machine learning techniques. Trans. Inst. Meas. Control..

[14] Drolc A., Vrtovšek J. (2010). Nitrate and nitrite nitrogen determination in waste water using on-line UV spectrometric method. Bioresour. Technol..

[15] Korshin G.V., Sgroi M., Ratnaweera H. (2018). Spectroscopic surrogates for real time monitoring of water quality in wastewater treatment and water reuse. Curr. Opin. Environ. Sci. Heal..

[16] Hoppe H., Messmann S., Giga A., Gruening H. (2011). A real-time control strategy for separation of highly polluted storm water based on UV–Vis online measurements—From theory to operation. Water Sci. Technol..

[17] Brito R.S., Pinheiro H.M., Ferreira F., Matos J.S., Lourenço N.D. (2013). In situUV-Vis spectroscopy to estimate COD and TSS in wastewater drainage systems. Urban Water J..

[18] Launay M.A., Dittmer U., Steinmetz H. (2016). Organic micropollutants discharged by combined sewer overflows–characterisation of pollutant sources and stormwater-related processes. Water Res..

[19] Mesquita D., Quintelas C., Amaral A.L.P.D., Ferreira E.C. (2017). Monitoring biological wastewater treatment processes: Recent advances in spectroscopy applications. Rev. Environ. Sci. Bio/Technol..

[20] Fleischmann N., Langergraber G., Weingartner A., Hofstaedter F., Nusch S., Maurer P. On-Line and in-Situ Measurement of Turbidity and COD in Wastewater Using UV/VIS Spectrometry. https://www.s-can.at/.

[21] Hochedlinger M., Hofbauer P., Wandl G., Meyer S., Rauch W., Kroiss H., Heindl M. Online UV-Vis measurements–The basis for future pollution based sewer real time control in Linz. Proceedings of the 2nd International IWA Conference on Sewer Operation and Maintenance.

[22] Van den Broeke J. (2007). On-line and in-situ UV/vis spectroscopy. AWE Int..

[23] Gruber G., Bertrand-Krajewski J.L., Beneditis J.D., Hochedlinger M., Lettl W. (2006). Practical aspects, experiences and strategies by using UV/VIS sensors for long-term sewer monitoring. Water Pract. Technol..

[24] Sarraguça M.C., Paulo A., Alves M.M., Dias A., Lopes J., Ferreira E.C. (2009). Quantitative monitoring of an activated sludge reactor using on-line UV-visible and near-infrared spectroscopy. Anal. Bioanal. Chem..

[25] Fogelman S., Zhao H., Blumenstein M., Zhang S. Estimation of oxygen demand levels using UV–vis spectroscopy and artificial neural networks as an effective tool for real-time, wastewater treatment control. Proceedings of the 1st Australian Young Water Professionals Conference.

[26] Del Río Cambeses H. (2011). Estudio de los Flujos de Contaminación Movilizados en Tiempo de Lluvia y Estrategias de Gestión en un Sistema de Saneamiento y Drenaje unitario de una Cuenca Urbana Densa de la España Húmeda. Ph.D. Thesis.

[27] García J.T., Espín-Leal P., Vigueras-Rodriguez A., Castillo L.G., Carrillo J.M., Martinez-Solano P., Nevado-Santos S. (2017). Urban Runoff Characteristics in Combined Sewer Overflows (CSOs): Analysis of Storm Events in Southeastern Spain. Water.

[28] García J.T., Espín-Leal P., Vigueras-Rodriguez A., Carrillo J.M., Castillo L.G. (2018). Synthetic Pollutograph by Prediction Indices: An Evaluation in Several Urban Sub-Catchments. Sustainability.

[29] Carreres-Prieto D., García J.T., Cerdán-Cartagena F., Suardíaz J. (2019). Spectroscopy Transmittance by LED Calibration. Sensors.

[30] Benavides M., Mailier J., Hantson A.-L., Muñoz G., Vargas A., Van Impe J.F., Wouwer A.V. (2015). Design and Test of a Low-Cost RGB Sensor for Online Measurement of Microalgae Concentration within a Photo-Bioreactor. Sensors.

[31] Carreres-Prieto D., García J.T., Cerdán-Cartagena F., Suardíaz J. (2020). Performing Calibration of Transmittance by Single RGB-LED within the Visible Spectrum. Sensors.

[32] Bozhynov V., Soucek P., Barta A., Urbanova P., Bekkozhayeva D. (2018). Visible Aquaphotomics Spectrophotometry for Aquaculture Systems. Proceedings of the Agreement Technologies.

[33] Wego A. (2013). Accuracy simulation of an LED based spectrophotometer. Optik.

[34] Schnable J.G., Grochowski P.J., Wilhelm L., Harding C., Kiefer M., Orr R.S. (1998). Portable LED-array VIS–NIR spectrophotometer/nephelometer. Field Anal. Chem. Technol..

[35] Rocha F.R.P., Martelli P.B., Reis B.F. (2004). Simultaneous in-line concentration for spectrophotometric determination of cations and anions. J. Braz. Chem. Soc..

[36] Venugopalan H. (2015). UVC LEDs enable cost-effective spectroscopic instruments. Laser Focus World.

[37] De la Torre C., Muñiz R., Pérez M.A. A new, low-cost, on-line RGB colorimeter for wine industry based on optical fibers. Proceedings of the XIX IMEKO World Congress.

[38] Sampedro Ó., Salgueiro J.R. (2015). Turbidimeter and RGB sensor for remote measurements in an aquatic medium. Measurements.

[39] Lima M.B., Andrade S.I., Neta M.S.S., Barreto I.S., Almeida L.F., De Araújo M.C.U. (2014). A Micro-Flow-Batch Analyzer using Webcam for Spectrophotometric Determination of Ortho -phosphate and Aluminium(III) in Tap Water. J. Braz. Chem. Soc..

[40] Suzuki Y., Aruga T., Kuwahara H., Kitamura M., Kuwabara T., Kawakubo S., Iwatsuki M. (2004). A simple and portable colorimeter using a red-green-blue light-emitting diode and its application to the on-site determination of nitrite and iron in river-water. Anal. Sci..

[41] SEOH Standard Cuvette Polystyrene Macro 3.5mL. UNSPSC Code: 41121813. https://uedata.amazon.com/SEOH-Standard-Cuvette-Polystyrene-Macro/dp/B00T5A64PQ.

[42] Field A. (2013). Discovering Statistics Using IBM SPSS Statistics.

[43] Osborne J. (2010). Improving your data transformations: Applying the Box-Cox transformation. Pract. Assess. Res. Eval..

[44] Mundry R., Nunn C.L. (2009). Stepwise Model Fitting and Statistical Inference: Turning Noise into Signal Pollution. Am. Nat..

[45] Abadi M., Barham P., Chen J., Chen Z., Davis A., Dean J., Kudlur M. Tensorflow: A system for large-scale machine learning. Proceedings of the 12th {USENIX} Symposium on Operating Systems Design and Implementation ({OSDI} 16).

[46] Oliphant T.E. A Guide to NumPy. https://ecs.wgtn.ac.nz/foswiki/pub/Support/ManualPagesAndDocumentation/numpybook.pdf.

[47] Van Der Walt S.J., Colbert S.C., Varoquaux G. (2011). The NumPy Array: A Structure for Efficient Numerical Computation. Comput. Sci. Eng..

[48] https://gplearn.readthedocs.io/en/stable/.

[49] Angeline P.J. (1997). Subtree crossover: Building block engine or macromutation. Genet. Program..

[50] Poli R. (2001). General Schema Theory for Genetic Programming with Subtree-Swapping Crossover. Proceedings of the Computer Vision.

[51] Harik G., Lobo F.G., Goldberg D. (1999). The compact genetic algorithm. IEEE Trans. Evol. Comput..

[52] Augusto D.A., Barbosa H.J.C. Symbolic regression via genetic programming. Proceedings of the Sixth Brazilian Symposium on Neural Networks.

[53] Vladislavleva E. (2008). Model-Based Problem Solving through Symbolic Regression via Pareto Genetic Programming.

[54] Back T. Optimal mutation rates in genetic search. Proceedings of the Fifth International Conference on Genetic Algorithms.

[55] Hagan M.T., Demuth H.B., Beale M. Neural Network Design.

[56] Osowski S. (1996). Sieci Neuronowe W Ujęciu Algorytmicznym.

[57] Coutsias E., Seok C., Dill K.A. (2004). Using quaternions to calculate RMSD. J. Comput. Chem..

[58] http://espectrofotometro.ingeniatic.com/.

[59] Murphy K., Heery B., Sullivan T., Zhang D., Paludetti L., Lau K.T., Diamond D., Costa E., O’Connor N.E., Regan F. (2015). A low-cost autonomous optical sensor for water quality monitoring. Talanta.

[60] Parra L., Rocher J., Escrivá J., Lloret J. (2018). Design and development of low cost smart turbidity sensor for water quality monitoring in fish farms. Aquac. Eng..

[61] Lambrou T.P., Anastasiou C.C., Panayiotou C.G., Polycarpou M.M. (2014). A Low-Cost Sensor Network for Real-Time Monitoring and Contamination Detection in Drinking Water Distribution Systems. IEEE Sens. J..

[62] Menon G.S., Ramesh M.V., Divya P. A low cost wireless sensor network for water quality monitoring in natural water bodies. Proceedings of the 2017 IEEE Global Humanitarian Technology Conference (GHTC).

[63] S: Scan Devide. https://www.s-can.at/es.

[64] Von Sperling M. (2015). Wastewater Characteristics, Treatment and Disposal. Water Intell. Online.

[65] Eriksson E., Auffarth K., Henze M., Ledin A. (2002). Characteristics of grey wastewater. Urban. Water.

[66] Bustillo-Lecompte C., Mehrvar M. (2015). Slaughterhouse wastewater characteristics, treatment, and management in the meat processing industry: A review on trends and advances. J. Environ. Manag..

[67] Eremektar G., Selçuk H., Meric S. (2007). Investigation of the relation between COD fractions and the toxicity in a textile finishing industry wastewater: Effect of preozonation. Desalination.

[68] Güngör-Demirci G., Demirer G.N. (2004). Effect of initial COD concentration, nutrient addition, temperature and microbial acclimation on anaerobic treatability of broiler and cattle manure. Bioresour. Technol..

[69] Hwang Y., Lee J.-K., Lee C., Jung Y., Cheong S., Lee C., Ku B., Jang S. (2007). Stability and thermal conductivity characteristics of nanofluids. Thermochim. Acta.

[70] Luo J., Ding L. (2011). Influence of pH on treatment of dairy wastewater by nanofiltration using shear-enhanced filtration system. Desalination.

[71] Papadopoulos A., Parissopoulos G., Papadopoulos F., Karteris A. Variations of COD/BOD5 ratio at different units of a wastewater stabilization pond pilot treatment facility. Proceedings of the 7th International Conference on Environmental Science and Technology Ermoupolis.

[72] Golfinopoulos S.K., Arhonditsis G.B. (2002). Multiple regression models: A methodology for evaluating trihalomethane concentrations in drinking water from raw water characteristics. Chemosphere.

[73] Cho J., Sung K.S., Ha S.R. (2004). A river water quality management model for optimising regional wastewater treatment using a genetic algorithm. J. Environ. Manag..

[74] Myers R.H., Myers R.H. (1990). Classical and Modern Regression with Applications.

[75] Zellner A., Chetty V.K. (1965). Prediction and decision problems in regression models from the Bayesian point of view. J. Am. Stat. Assoc..

[76] Bottenberg R.A., Ward J.H. (1963). Applied multiple linear regression. PsycEXTRA Dataset.

[77] Zhang M., Tay J.H., Qian Y., Gu X.S. (1998). Coke plant wastewater treatment by fixed biofilm system for COD and NH3-N removal. Water Res..

[78] Brookman S. (1997). Estimation of biochemical oxygen demand in slurry and effluents using ultra-violet spectrophotometry. Water Res..

[79] Chen H., Shi J.-L., Yang Y., Li Y., Yan D.-S., Shi C.-S. (2002). Violet-blue photoluminescent properties of mesoporous zirconia modified with phosphoric acid. Appl. Phys. Lett..

[80] Fredin L., Nelander B., Ribbegård G. (1977). Infrared spectrum of the water dimer in solid nitrogen. I. Assignment and force constant calculations. J. Chem. Phys..

[81] Dalal R.C., Henry R.J. (1986). Simultaneous Determination of Moisture, Organic Carbon, and Total Nitrogen by Near Infrared Reflectance Spectrophotometry. Soil Sci. Soc. Am. J..

[82] Reeves J.B., Van Kessel J.S. (2000). Near-infrared spectroscopic determination of carbon, total nitrogen, and ammonium-N in dairy manures. J. Dairy Sci..

[83] Shi T., Cui L., Wang J., Fei T., Chen Y., Wu G. (2012). Comparison of multivariate methods for estimating soil total nitrogen with visible/near-infrared spectroscopy. Plant. Soil.

