# Supplementary material for: Wastewater Quality Estimation through Spectrophotometry-Based Statistical Models
Source: Sensors (Basel). 2020 Oct 1;20(19):5631. doi: 10.3390/s20195631 (PMC7582758; doi:10.3390/s20195631)
Supplement: Supplementary file 1 [file sensors-20-05631-s001.pdf]

sensors

Supplementary Materials

Article

Wastewater Quality Estimation Through Spectrophotometry-Based Statistical Models

Daniel Carreres-Prieto [1,*], Juan T. García [1,*], Fernando Cerdán-Cartagena [2] and Juan Suardiaz-Muro [3]

[1] Department of Mining and Civil Engineering, Universidad Politécnica de Cartagena, City post code, Country
[2] Department of Information and Communications Technologies, Universidad Politécnica de Cartagena, City post code, Country; fernando.cerdan@upct.es
[3] Department of Electronic Technology, Universidad Politécnica de Cartagena, City post code, Country; juan.suardiaz@upct.es
* Correspondence: daniel.carreres@upct.es (D.C.-P.); juan.gbermejo@upct.es (J.T.G.)

Received: 23 August 2020; Accepted: 29 September 2020; Published: 1 October 2020

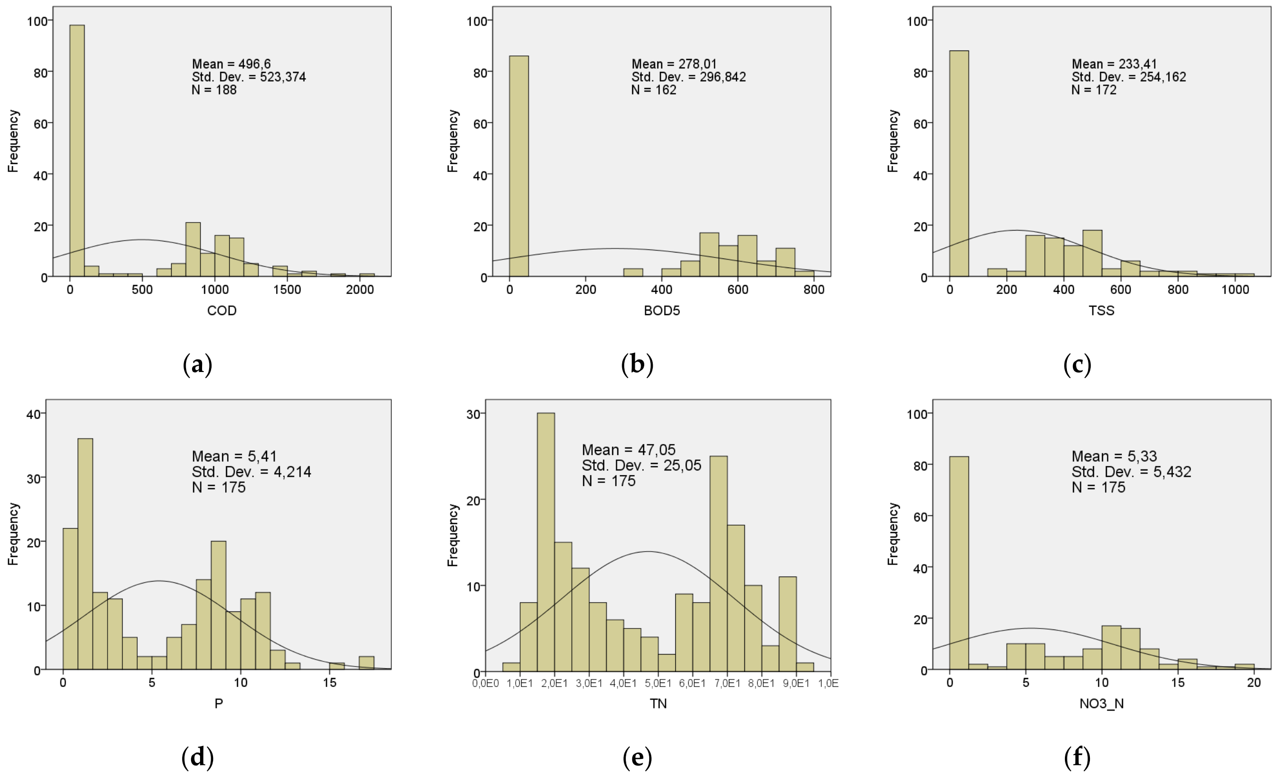

Figure S1. Histogram of combined raw and treated water samples. (**a**) COD, (**b**) BOD5, (**c**) TSS, (**d**) P, (**e**) TN and (**f**) NO_3^-N.

Table S1. Basic characteristics of raw water data sets for MLR study.

Parameter	Total Samples	Outliers	Valid Samples	Normality Test	
				Kolmogorov-Smirnov	Shapiro-Wilk
COD	108	7	101	0.2*	0.376
BOD_5	108	22	86	0.2*	0.172
TSS	108	16	92	0.2*	0.043
P	108	2	106	0.2*	0.468
PH	108	1	107	0.2*	0.715
NO3-N	108	11	97	0	0

* This is a lower bound of the true significance.

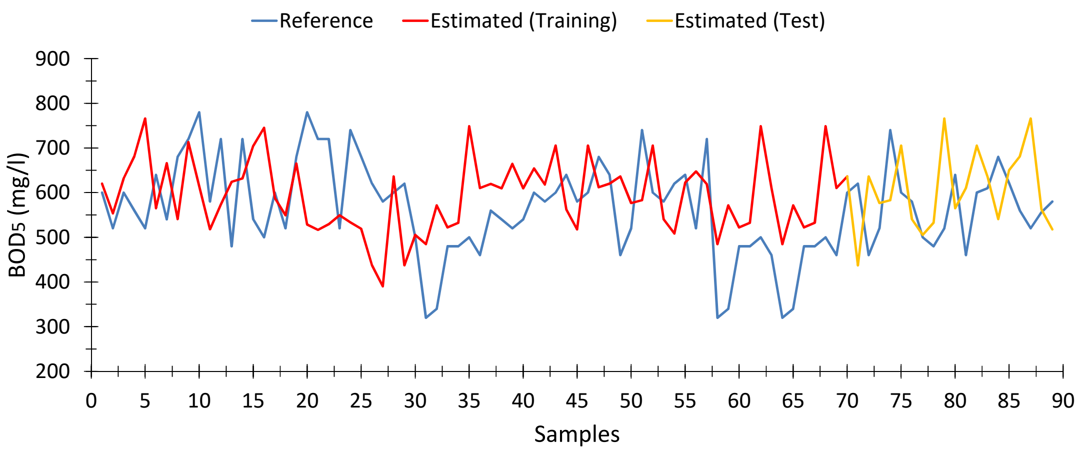

Figure S2. Comparison between BOD_5 values measured by the wastewater treatment plant and the values calculated from spectrophotometric data by multivariate linear regression model, for wastewater (raw water).

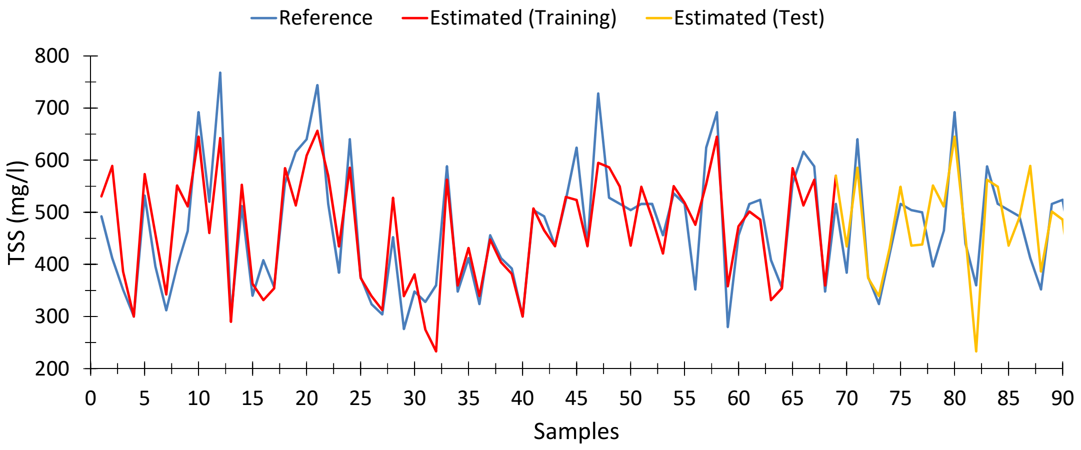

Figure S3. Comparison between TSS values measured by the wastewater treatment plant and the values calculated from spectrophotometric data by multivariate linear regression model, for wastewater (raw water).

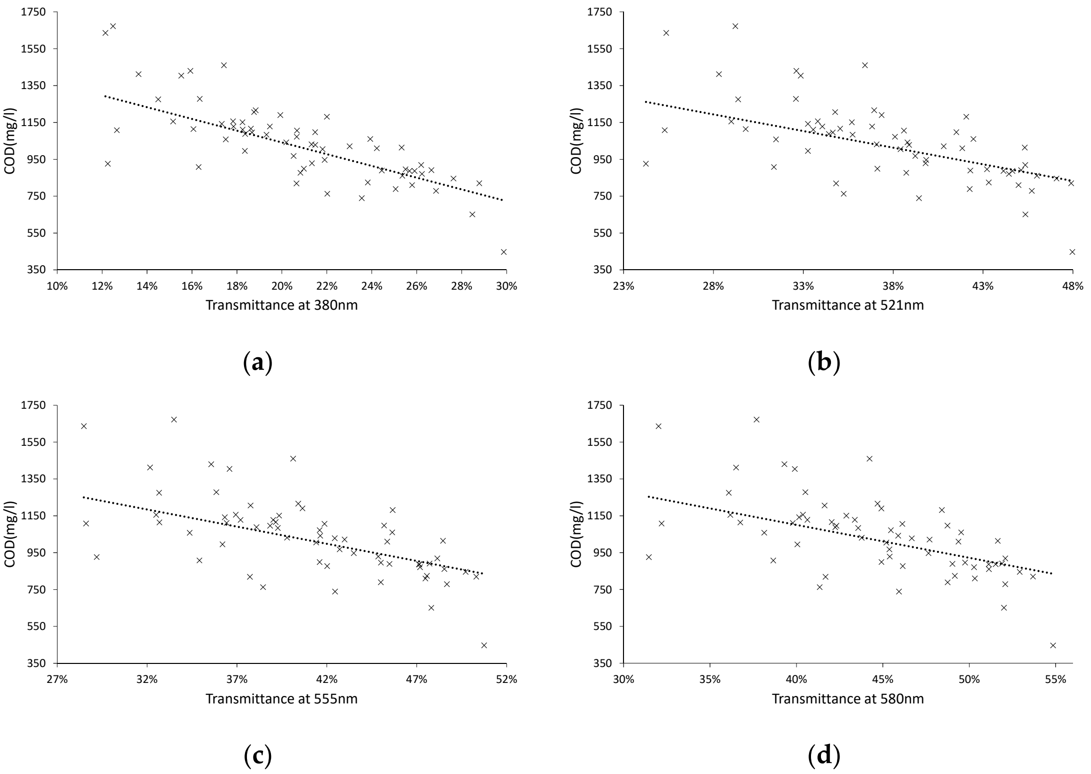

Figure S4. Scatter plot of the variables used in the MLR model for calculating COD with respect to: (**a**) Transmittance at 380 nm, (**b**) Transmittance at 521 nm, (**c**) Transmittance at 555 nm and (**d**) Transmittance at 580 nm.

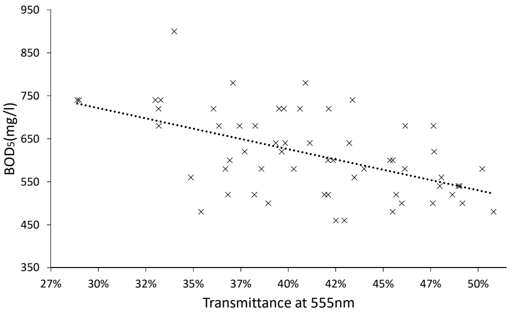

(**a**)

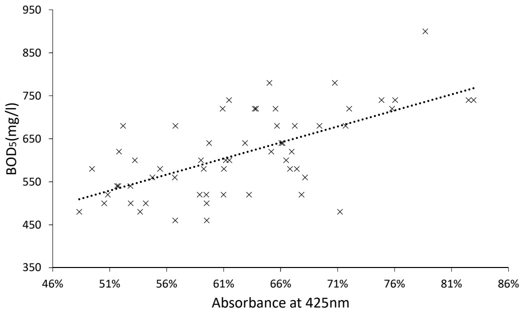

(**b**)

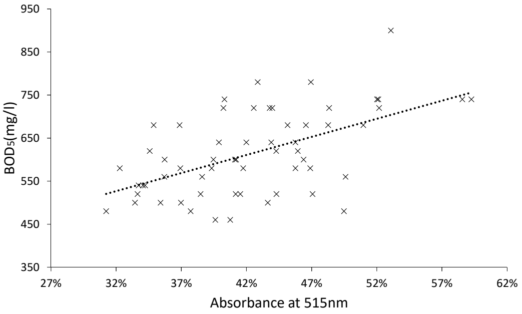

(**c**)

Figure S5. Scatter plot of the variables used in the MLR model for calculating BOD_5 with respect to: (**a**) Transmittance at 555 nm, (**b**) Absorbance at 425 nm and (**c**) Absorbance at 515 nm.

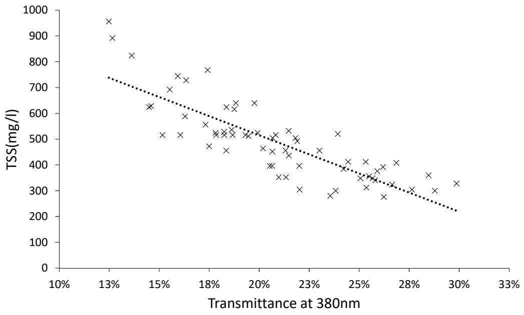

(**a**)

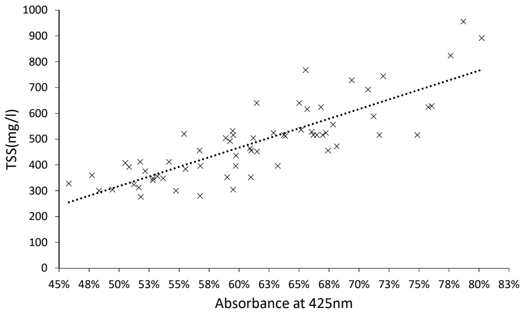

(**b**)

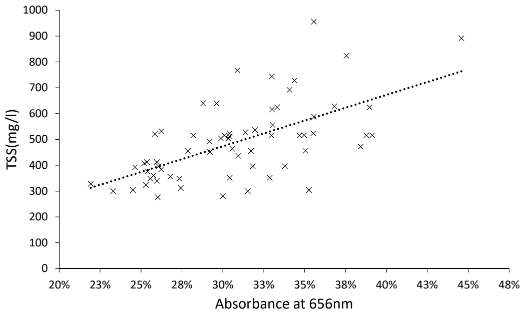

(**c**)

Figure S6. Scatter plot of the variables used in the MLR model for calculating TSS with respect to: (**a**) Transmittance at 380 nm, (**b**) Absorbance at 425 nm and (**c**) Absorbance at 655 nm.

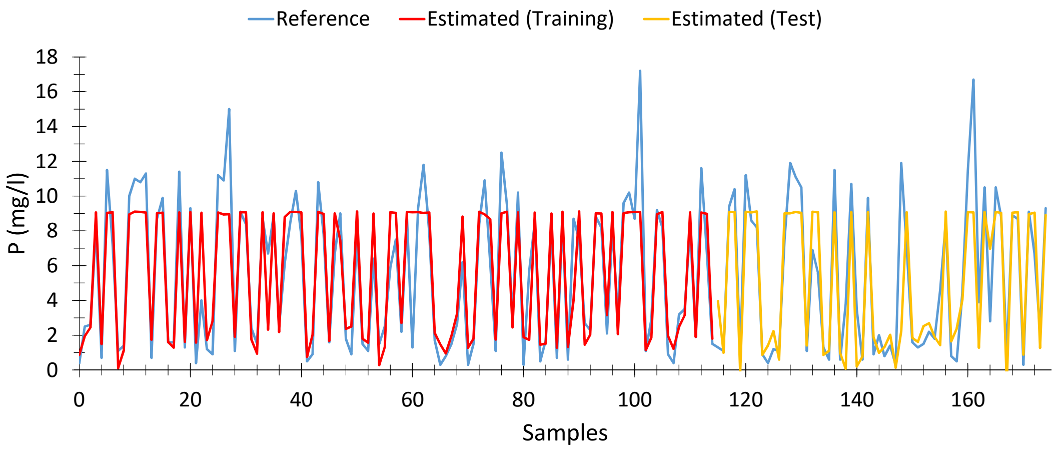

Figure S7. Comparison between P values measured by the wastewater treatment plant and the values calculated from spectrophotometric data, according to Equation 9.

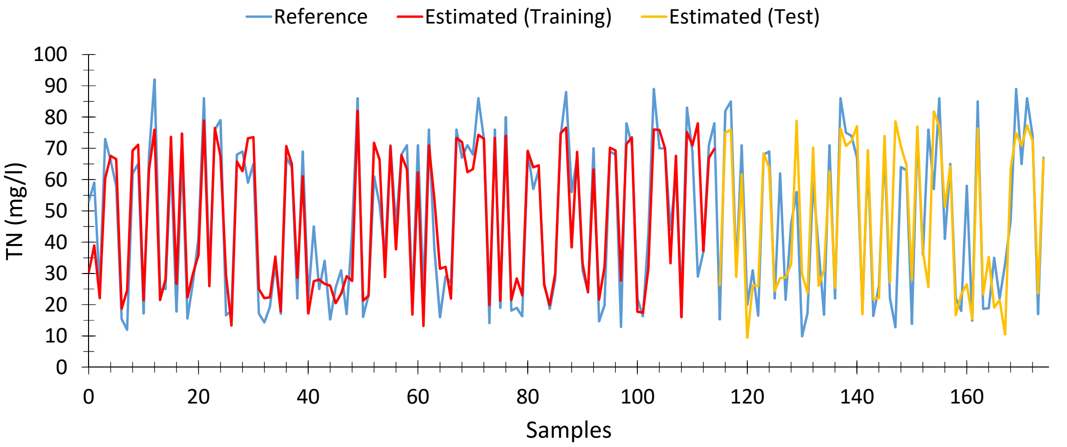

Figure S8. Comparison between TN values measured by the wastewater treatment plant and the values calculated from spectrophotometric data, according to Equation 10.

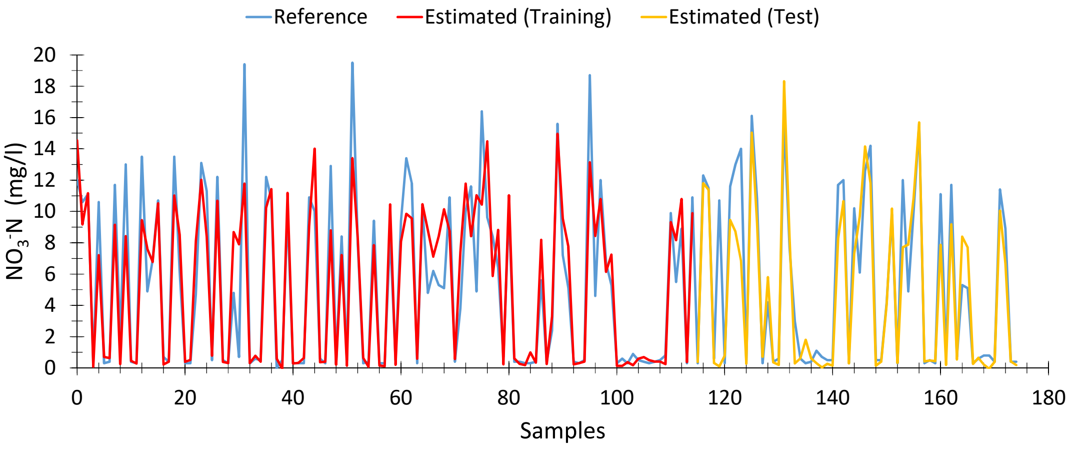

Figure S9. Comparison between NO_3^-N values measured by the wastewater treatment plant and the values calculated from spectrophotometric data, according to Equation 11.
